# A Mineral-Doped Micromodel Platform Demonstrates Fungal Bridging of Carbon Hot Spots and Hyphal Transport of Mineral-Derived Nutrients

**DOI:** 10.1128/msystems.00913-22

**Published:** 2022-11-17

**Authors:** Arunima Bhattacharjee, Odeta Qafoku, Jocelyn A. Richardson, Lindsey N. Anderson, Kaitlyn Schwarz, Lisa M. Bramer, Gerard X. Lomas, Daniel J. Orton, Zihua Zhu, Mark H. Engelhard, Mark E. Bowden, William C. Nelson, Ari Jumpponen, Janet K. Jansson, Kirsten S. Hofmockel, Christopher R. Anderton

**Affiliations:** a Environmental Molecular Sciences Division, Earth and Biological Sciences Directorate, Pacific Northwest National Laboratory, Richland, Washington, USA; b Stanford Synchrotron Radiation Lightsource, SLAC National Accelerator Laboratory, Menlo Park, California, USA; c Biological Sciences Division, Earth and Biological Sciences Directorate, Pacific Northwest National Laboratory, Richland, Washington, USA; d Division of Biology, Kansas State University, Manhattan, Kansas, USA; e Department of Agronomy, Iowa State University, Ames, Iowa, USA; Tufts University

**Keywords:** secondary ion mass spectrometry, X-ray absorption, biomineralization, nutrient transport, proteomics

## Abstract

Soil fungi facilitate the translocation of inorganic nutrients from soil minerals to other microorganisms and plants. This ability is particularly advantageous in impoverished soils because fungal mycelial networks can bridge otherwise spatially disconnected and inaccessible nutrient hot spots. However, the molecular mechanisms underlying fungal mineral weathering and transport through soil remains poorly understood primarily due to the lack of a platform for spatially resolved analysis of biotic-driven mineral weathering. Here, we addressed this knowledge gap by demonstrating a mineral-doped soil micromodel platform where mineral weathering mechanisms can be studied. We directly visualize acquisition and transport of inorganic nutrients from minerals through fungal hyphae in the micromodel using a multimodal imaging approach. We found that Fusarium sp. strain DS 682, a representative of common saprotrophic soil fungus, exhibited a mechanosensory response (thigmotropism) around obstacles and through pore spaces (~12 μm) in the presence of minerals. The fungus incorporated and translocated potassium (K) from K-rich mineral interfaces, as evidenced by visualization of mineral-derived nutrient transport and unique K chemical moieties following fungus-induced mineral weathering. Specific membrane transport proteins were expressed in the fungus in the presence of minerals, including those involved in oxidative phosphorylation pathways and the transmembrane transport of small-molecular-weight organic acids. This study establishes the significance of a spatial visualization platform for investigating microbial induced mineral weathering at microbially relevant scales. Moreover, we demonstrate the importance of fungal biology and nutrient translocation in maintaining fungal growth under water and carbon limitations in a reduced-complexity soil-like microenvironment.

**IMPORTANCE** Fungal species are foundational members of soil microbiomes, where their contributions in accessing and transporting vital nutrients is key for community resilience. To date, the molecular mechanisms underlying fungal mineral weathering and nutrient translocation in low-nutrient environments remain poorly resolved due to the lack of a platform for spatial analysis of biotic weathering processes. Here, we addressed this knowledge gap by developing a mineral-doped soil micromodel platform. We demonstrate the function of this platform by directly probing fungal growth using spatially resolved optical and chemical imaging methodologies. We found the presence of minerals was required for fungal thigmotropism around obstacles and through soil-like pore spaces, and this was related to fungal transport of potassium (K) and corresponding K speciation from K-rich minerals. These findings provide new evidence and visualization into hyphal transport of mineral-derived nutrients under nutrient and water stresses.

## INTRODUCTION

Biotic mineral weathering is critical in impoverished soils where nutrients can be bound to minerals and unavailable to plants and other organisms. Some soil fungi are adept at extraction of inorganic nutrients from soil minerals by weathering rock material to mineralize elemental potassium (K), iron (Fe), manganese (Mn), calcium (Ca), and other inorganic nutrients (1–3). These fungi release elemental nutrients from rock-derived minerals either (i) indirectly, by secreting low-molecular-weight organic acids into the soil microenvironment, or (ii) directly, by exerting physical forces at the hypha-mineral interface (4). Some mycorrhizal fungi then transfer the mineral-derived nutrients to host plants in exchange for carbon (5–8). Several instances of both ectomycorrhizal and arbuscular mycorrhizal fungal weathering of P and K containing soil minerals have been documented, where the main mechanism of weathering is by dissolution with organic acids (9–13). Specifically, mycorrhizal associations with plants improve K uptake through fungus-induced mineral weathering and transport of K to their host plants (14–16). Saprotrophic fungi can also contribute to soil mineral weathering (17–21), although less is known about the underlying mechanisms compared to mycorrhizae. A study with the saprotrophic fungus Aspergillus niger found evidence of direct and indirect mineral weathering in liquid cultures containing mineral grains (22).

Widespread fungal hyphal networks on different soil mineral surfaces facilitate weathering of minerals and transport of mineral-derived nutrients through the complex soil landscape (23–25). Several studies have investigated how these fungal mycelial networks bridge carbon-rich nutrient hot spots in soil to translocated nutrient form such hot spots and from minerals (26, 27). However, fungal induced mineral weathering and nutrient transport is a spatially heterogeneous process in nature, and bulk soil measurements do not provide a fully representative understanding of fungal hyphal interactions at the mineral surface within soil systems. Moreover, the filamentous and microscopic nature of fungal hyphae requires investigation of mineral weathering at the hypha-mineral interface, and the ability to resolve weathering processes spatially at these microbial scales could provide more comprehensive insights into nutrient transport at the ecosystem scale. Further complicating the matter, diffusion of fungal exuded organic acids in saturated soils can dramatically alter the heterogeneity of mineral weathering processes (28). As such, the spatial heterogeneity of mineral weathering processes in, and the opacity of, soil presents unique challenges in experimentally identifying specific fungal interactions within the complex soil habitat. Consequently, mineral weathering and transport mechanisms in fungi have not been spatially resolved, in part due to the lack of analysis platforms that allow spatial visualization and analysis of fungal induced mineral weathering. At the bulk soil scale, fungal induced mineral weathering has been extensively studied in mycorrhizal fungi (9–11).

Here, we developed a novel mineral-doped soil micromodel platform that provided unprecedented spatial visualization into the fungal thigmotropism, mineral weathering, and nutrient transport by a soil saprotrophic fungus, Fusarium sp. DS 682 (29). One reason we used a saprotrophic fungus to demonstrate the micromodel platform is because culturing mycorrhizal fungus axenically is challenging due to their strong associations with plants (30). Nevertheless, the mineral doping technology demonstrated here is translatable to larger device designs for plant and fungal growth experiments. Using this platform, we show that this fungal species bridge carbon-rich hot spots, weather mineral interfaces, and transport inorganic nutrients through extraction of K by an indirect mineral weathering mechanism in an environment that mimics select soil physical and chemical properties. The results of this study provide a microscale level understanding of fungal transport of K^+^ from mineral interfaces, and K speciation in minerals after fungal growth.

## RESULTS

### Development of the mineral-doped soil micromodel platform for probing fungal induced mineral weathering.

**FIG 1:**
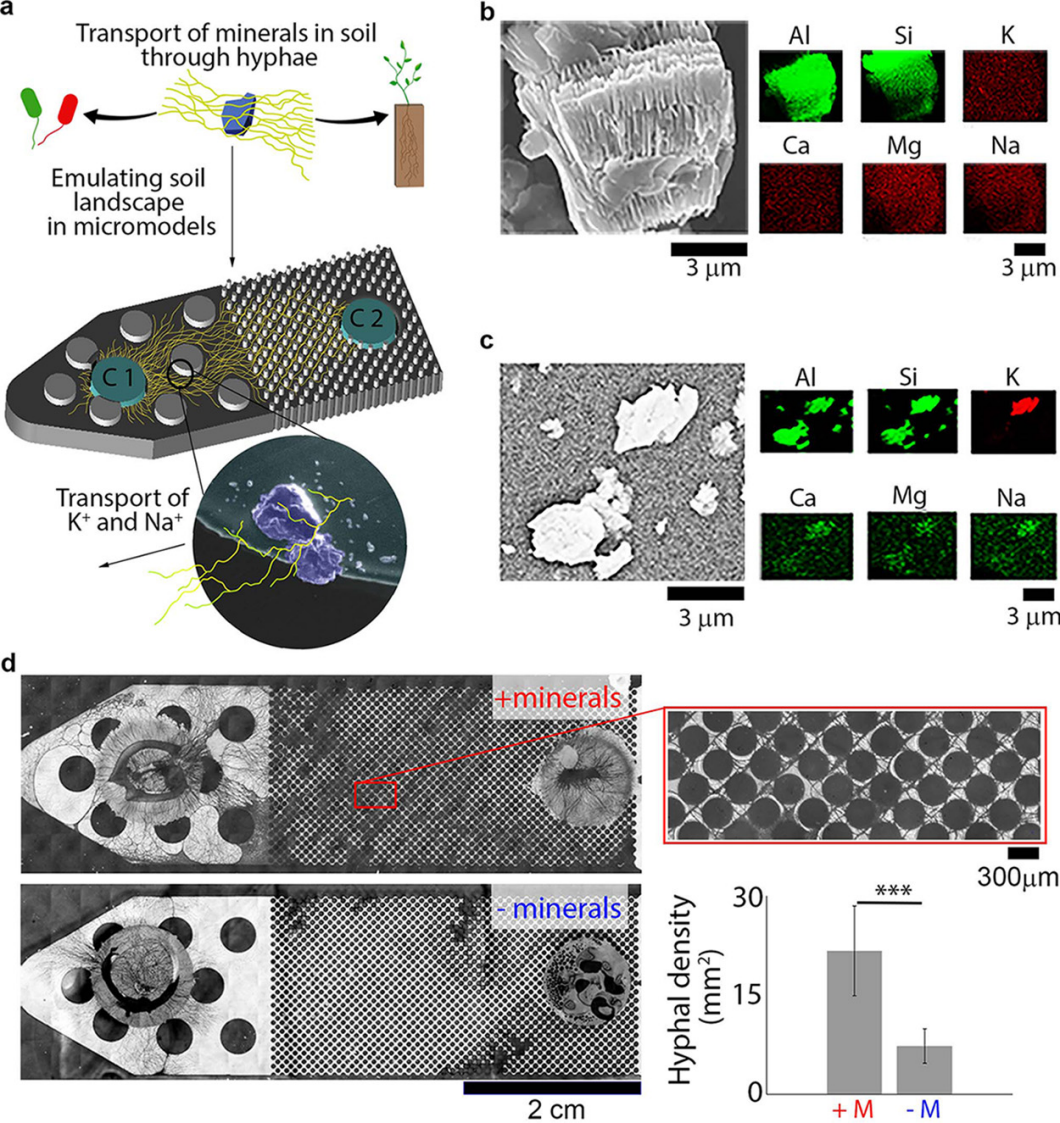
Fungal growth during water and nutrient limitation is contingent on the presence of soil minerals with accessible nutrients. (a) Schematic of the soil micromodel used for this study, which emulates natural soil pore spacing and mineralogy. Fungal growth in a water- and nutrient-limited environment is enhanced in mineral-doped micromodels due to the extraction of K^+^ and Na^+^ from minerals through mycelia. The PDA plugs—“C1” and “C2”—are the only nutrient sources present in the micromodel, and the fungus was inoculated onto C1. (b) Energy dispersive X-ray (EDX) images of the powdered minerals used to dope micromodels with minerals show the presence of essential elements, such as K, Ca, Mg, and Na, which could be used by the fungus for growth in this water- and nutrient-limited microenvironment. (c) EDX images of the same mineral powder embedded in the PDMS matrix, which demonstrates their accessibility in these micromodel systems. (d) PDMS mineral-doped micromodel with minerals (top, + minerals) shows enhanced fungal growth, whereas fungal growth is limited in PDMS micromodels without minerals (bottom, − minerals). We observed thigmotropic response in the mineral-doped micromodel shown in the zoomed-in image on the right within the red square. Quantitative fungal growth comparisons in nutrient-limited soil micromodels with (+M) or without (−M) minerals show a significant increase in hyphal density in the presence of minerals (bar graph, bottom right). ***, *P* < 0.0001. The image analysis method is described in Fig. S4.

We developed a mineral-doped soil micromodel (Fig. 1a) that simulates a weathered soil environment, where key nutrients (e.g., K, Fe, Ca, and Mn) were not readily bioavailable because of their strong associations to minerals. The soil micromodel features an evenly spaced matrix of pillars that mimics pore spacing found in the soil environment (31), and the device was fabricated with polydimethylsiloxane (PDMS) doped with minerals. To build the mineral-doped soil micromodel, we used a natural kaolinite powder that contained 7% K-containing feldspar (K-feldspar) and 4% mica components that are common minerals present within soil (32). Elemental analysis of the natural kaolinite showed an abundance of essential nutrients such as K, Na, Mg, and Ca (Fig. 1b), in addition to Al and Si. These essential nutrients likely originated from the K-feldspar and interlayer regions of phyllosilicates mica and kaolinite (see Fig. S1 in the supplemental material). The use of a natural mineral enabled interrogation of the release and transport of mineral nutrients by the fungal hyphae under controlled environmental conditions. The minerals embedded in the surface of the microchannel in the soil micromodel also provided a direct contact with the fungus so that this platform can be used to study both direct and indirect weathering mechanisms (Fig. 1c; see also Fig. S2a). In case of indirect weathering, fungi secrete organic acids that weather the mineral surface through dissolution, while fungal hyphae exert force to create tunnels on mineral surfaces through direct weathering. Both instances of direct and indirect weathering can be found in nature and often occurring simultaneously (4).

10.1128/msystems.00913-22.1 FIG S1 The natural kaolinite powder contained minerals with essential nutrients, which were still present once embedded in the micromodel. X-ray diffraction (XRD) analysis comparison of the natural kaolinite powder and the mineral embedded in the soil micromodels demonstrates the presence of minerals such as feldspar, mica, and quartz, along with kaolinite. The results also show that the mineral doping process within the PDMS does not alter presence of the mineral phases of the natural kaolinite powder. Download FIG S1, TIF file, 0.10 MB. https://doi.org/10.1128/AuthorWarrantyLicense.v1 This is a work of the U.S. Government and is not subject to copyright protection in the United States. Foreign copyrights may apply.

10.1128/msystems.00913-22.2 FIG S2 Use of dry etching process helps facilitate the access of minerals for fungi on the surface of the micromodel. (a) Schematic demonstrating that minerals embedded in the PDMS matrix are made available at the surface of the micromodel, for access to fungal mycelia, through deep reactive ion etching (DRIE). We observed that fungal growth is limited when minerals are not made available at the surface. (b) Secondary ion images of the mineral-doped micromodel surface before (top-row images) and after DRIE (bottom-row images). The surface analysis of the micromodels before and after etching demonstrates that K and Al is more available on the surface after etching. (c) X-ray photoelectron spectroscopy analysis of mineral grains etched by the DRIE process shows binding energy peak shifts of Al 2p corresponding to the formation of AlF_3_. However, peak shifts related to K (i.e., K 2p_1/2_ and K 2p_3/2_) were not observed, indicating the K in the mineral grains remains unchanged after the etching process. These results suggest that any observed mineral speciation will be from biotic mineral degradation by the fungi and not from abiotic micromodel fabrication processes. The table on the right shows atomic percentages of different elements in etched and unetched mineral grains, where the fluorine content is increased in etched mineral due to etching by SF_6_ plasma. The K content is comparable in both etched and unetched minerals. Download FIG S2, JPG file, 0.7 MB. https://doi.org/10.1128/AuthorWarrantyLicense.v1 This is a work of the U.S. Government and is not subject to copyright protection in the United States. Foreign copyrights may apply.

10.1128/msystems.00913-22.4 FIG S4 Image analysis workflow demonstrates differences in hyphal density in micromodels with or without minerals. The images demonstrate the workflow using ImageJ software to quantify the hyphal density (see Fig. 1d) from optical images. Ten images were used per condition, and we show three different images per condition here to demonstrate the progression of the image analysis to obtain hyphal density of fungal growth with or without minerals. Download FIG S4, JPG file, 0.1 MB. https://doi.org/10.1128/AuthorWarrantyLicense.v1 This is a work of the U.S. Government and is not subject to copyright protection in the United States. Foreign copyrights may apply.

The soil micromodel was inoculated with Fusarium sp. strain DS 682, a saprotrophic fungus that is common in grassland soils (29). The fungus was inoculated at one end with a potato dextrose agar (PDA) plug (Fig. 1a, location C1), while a second axenic PDA patch (Fig. 1a, location C2) was provided at the other end of the device. The PDA plugs were not in contact with the minerals within the microchannel throughout fungal growth and only served as two carbon-rich hot spots (C1 and C2) at the two ends of the microchannel. The mineral grains embedded in the micromodel were the only source of nutrients, such as K, Na, and Ca (Fig. 1a) between the carbon-rich PDA plugs (C1 and C2) at either end of the device, creating a carbon-impoverished condition within the microchannel. Fungus-driven weathering of the minerals was thus required for making the mineral-derived inorganic nutrients available for hyphal growth. A water limitation stress was simulated in the soil micromodel by creating an unsaturated channel to keep the PDA sources apart (C1 and C2), preventing diffusion of carbon between the PDA plugs. The water stress also prevented diffusion of mineral-derived nutrients (Fig. 1a) through liquid media in the microchannel; however, some diffusion might have occurred through fungal exudation. In this experimental design, fungal contact with minerals was required to induce weathering of mineral surfaces for the extraction of nutrients because the water stress induced in the microchannel resulted in limited diffusion of fungal organic acids. Therefore, the fungal hyphae likely weathered mineral particles locally, where organic acids were exuded. A micromodel without minerals, but with PDA plugs and fungi inoculated at C1, served as a control in our experiments.

### Mineral availability regulates fungal growth in nutrient-limited environments.

The micromodel system provided an unprecedented view of fungal mineral weathering mechanisms using several spatially resolved characterization techniques, such as optical microscopy, electron microscopy, secondary ion mass spectrometry, and X-ray spectromicroscopy. The PDMS-glass configuration of the micromodels was reversibly bonded such that the PDMS mineral micromodel side could be separated from the glass coverslip for characterizing fungal mineral weathering using advanced imaging techniques.

We used optical microscopy to image the growth of the fungal hyphae from the inoculation site (C1) through the porous environment (12- to 150-μm pore spaces) to access the axenic nutrient (PDA) pool (Fig. 1a, C2). We found that fungal mycelia grew extensively bridging C1 and C2 in the presence of minerals and exhibited thigmotropic movement around micro- and macropore spaces that mimicked natural soil pores (Fig. 1d). In contrast, the absence of soil minerals in the positive-control micromodel significantly inhibited fungal growth (Fig. 1d), where fungi grew only around the nutrient plug at the inoculation site (C1). Further tests suggest that this increase in hyphal density in microporous environments mimicking soil were specifically regulated by the fungal-mineral interactions. Notably, the porosity of the micromodels without minerals did not significantly alter hyphal growth, and fungal hyphal biomass was observed in micromodels with larger pore spaces (600 μm). However, growth ceased when the pore size decreased to 5 μm (see Fig. S3a), demonstrating that thigmotropic movement was at least partly dependent on pore size. In addition, thigmotropic movement could not be attributed to the surface chemistry of the PDMS, since micromodels with either hydrophilic or hydrophobic surfaces resulted in fungal growth comparable to micromodels without minerals (see Fig. S3b). Combined, these results illustrate the role of mineral-derived inorganic nutrients in fungal hyphal thigmotropic movement within microporous environments mimicking soil.

10.1128/msystems.00913-22.3 FIG S3 Fungal hyphal growth is dependent on pore size within micromodels but not on the surface chemistry of the PDMS. (a) Optical images of fungal growth in micromodels with different porosities shows hyphal growth can occur through bigger pore sizes (600 μm), but growth is attenuated in pore spaces closer to those observed in soil (5 μm). (b) Fungal hyphal growth is not dependent on the surface chemistry of soil micromodels, as shown by the similar growth behavior that was observed in micromodel with PDMS interfaces that were hydrophilic and hydrophobic. Download FIG S3, JPG file, 0.1 MB. https://doi.org/10.1128/AuthorWarrantyLicense.v1 This is a work of the U.S. Government and is not subject to copyright protection in the United States. Foreign copyrights may apply.

### Direct visualization of fungal nutrient uptake from minerals and K speciation by fungal hyphae.

**FIG 2:**
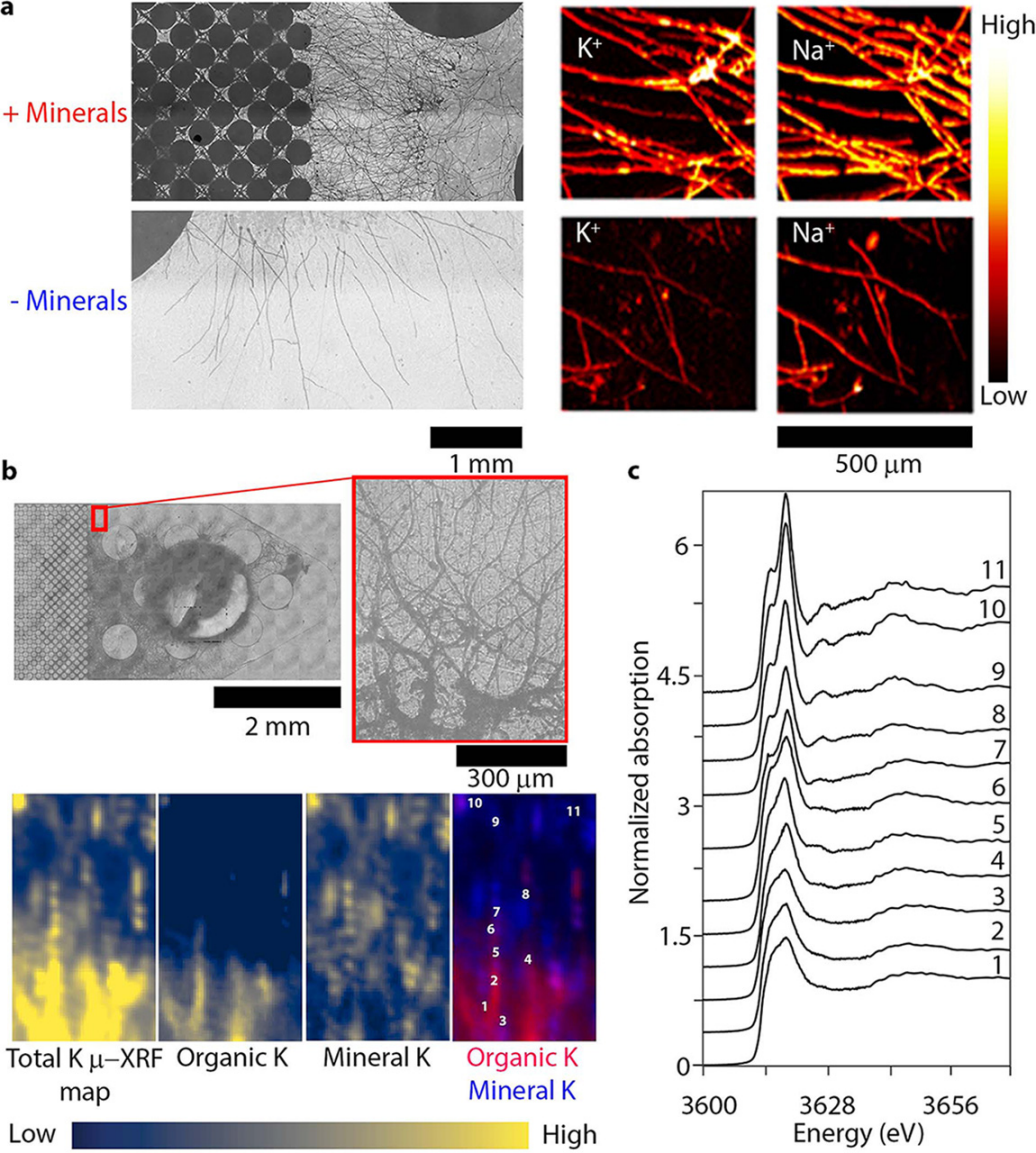
Direct evidence of fungal hyphal uptake and organic complexation of essential nutrients from degrading minerals in soil micromodels. (a) Optical microscopy images of fungal growth (left) and time-of-flight secondary ion mass spectrometry (ToF-SIMS) analysis (right) of fungal hyphae grown in micromodels with or without minerals. ToF-SIMS results demonstrate an increased uptake of K^+^ and Na^+^ ions along fungal hyphae in micromodels containing minerals in comparison to those grown in a mineral-less environment. (b) Optical image of mineral-doped micromodel showing the μ-XRF mapping region in the red box to the corresponding μ-XRF maps. The four lower panels show, from left to right, maps of total K (above the K K-edge at 3,627 eV), organic complexed K, mineral-bound K, and a dual-color plot of organic complexed K (red) and mineral-bound K (blue) with numbers corresponding to the locations of XANES spectra in panel c. Maps of organic K and mineral K were created by fitting multienergy maps with end-member spectra. The hypha-rich region in the optical image corresponds to organic complexed K in the XRF maps and shows different forms of K exists in the micromodel, as a result of fungal growth. (c) XANES spectra from the locations numbered in XRF maps in panel b. The spectra show a change from organic K (1 to 6) dominated to mineral-bound K (7 to 11) across the mapped region. In spectra representing mineral-bound K (7 to 11), there are shifts in the peak energy, demonstrating changes in K mineral chemistries precipitated from fungal degradation of minerals in the micromodel surface. The linear combination fitting of spectra 1 to 11 showing the percentages of mineral and organic complexed K is shown in Fig. S7.

We observed an increase in the relative concentrations of K^+^ and Na^+^ in fungal hyphae grown in mineral-doped soil micromodels compared to mineral free micromodel controls by time-of-flight secondary ion mass spectrometry (ToF-SIMS) imaging (Fig. 2a). We used PDA that contained minimal amounts of K and Na because there was no fungal growth in minimal media without any source of Na and K. The micromodels with or without minerals had the same amount of PDA plugs, which contained minimal amounts of K and Na. Therefore, any increase in relative concentrations of K^+^ and Na^+^ in the fungal hyphae in the mineral-doped micromodels was attributed to uptake from the K-feldspar, mica minerals, or K from interlayer regions of the phyllosilicates. We did not observe an increase in other nutrients present within mineral grains shown in Fig. 1b and c (e.g., Ca and Mg) in the fungal hyphae grown in the presence of minerals compared to the absence of minerals.

10.1128/msystems.00913-22.7 FIG S7 K speciation occurs along with fungal growth in a mineral-doped micromodel. The linear combination fitting of the XANES spectra from the locations numbered in Fig. 2c. Spectra show a change from organic K (numbers 1 to 6) dominated to mineral bound K (numbers 7 to 11) across the mapped region (left). The linear combination fitting using organic complexed spectra shows different percentages of mineral and organic complexed K on the micromodel corresponding to fungal hyphae and speciated minerals in soil micromodels (right). Download FIG S7, JPG file, 1.1 MB. https://doi.org/10.1128/AuthorWarrantyLicense.v1 This is a work of the U.S. Government and is not subject to copyright protection in the United States. Foreign copyrights may apply.

We hypothesized that enhanced fungal growth in the presence of minerals was due to the uptake of nutrients by hyphae from minerals in the micromodel through mineral weathering by the release of fungal organic acids. Consequently, we expected to observe differences in K chemistry in mineral interfaces and K^+^ transported in fungal hyphae from K minerals. Using multi-energy micro-X-ray fluorescence imaging (μ-XRF) around the potassium K-edge, combined with potassium X-ray Absorption Near Edge Structure (XANES) spectroscopy, we found evidence of K^+^ transport from minerals through the fungal hyphae (Fig. 2b and c). Specifically, we identified two distinct K chemistries in the mineral-doped soil micromodel as a result of fungal growth: (i) inorganic mineral-bound K (referred to as “inorganic K”), and (ii) unidentified organic, possibly hyphal adsorbed, K (referred to as “organic K”) located along distinct paths (Fig. 2b). The distinct paths of the organic K coincided with elevated S and P abundances that are indicative of fungal hyphal biomass (see Fig. S5). The organic K was observed at the fungal inoculation point and in fungal hyphae grown with and without minerals, suggesting that this form of K is highly present in fungal hyphae.

10.1128/msystems.00913-22.5 FIG S5 The location of fungal hyphal growth was identified in μ-XRF maps by visualizing the locations of P and S. Optical images (top) of μ-XRF mapping region, with the specific region denoted by a red box and the associated zoomed in the image, and μ-XRF maps of organic associated K and mineral associated K (bottom, left), P (bottom, center), and S (bottom, right). Detection of P and S is associated with fungal hyphal biomass in the micromodels, as shown in the optical image of the same region as the XRF maps. Download FIG S5, JPG file, 0.1 MB. https://doi.org/10.1128/AuthorWarrantyLicense.v1 This is a work of the U.S. Government and is not subject to copyright protection in the United States. Foreign copyrights may apply.

The extensive fungus-induced K speciation of the minerals was probed using spatially defined XANES spectra within the soil micromodel (Fig. 2c). Here, mineral K showed the distinct pre- and postedge features (Fig. 2c, spectra 7 to 11), which were absent in the organic K spectra collected from fungal biomass growing in the same region (Fig. 2c, spectra 1 to 6). Interestingly, we observed peak shifts in energy of the spectral features within inorganic K spectra (Fig. 2c, spectra 7 to 11), while the peaks for organic K spectra (Fig. 2c, spectra 1 to 6) did not exhibit energy shifts. Mineral weathering by different fungus-derived organic acids resulting in distinctive mineral K bonding environments could contribute to these shifts in energy observed for the pre- and postedge features in inorganic K spectra (Fig. 2c; see also Fig. S7). In addition, a linear combination fitting of end-member organic K and mineral XANES spectra (see Fig. S6) to the experimental micromodel K XANES spectra indicates discrepancies in the fits of spectra 7 to 11, which alludes to a change in the mineral surface chemistry (see Fig. S7).

10.1128/msystems.00913-22.6 FIG S6 The K bonding environment vary widely between organic- and mineral-derived K. XANES spectra of the different end member controls (solid lines, top to bottom): fungal growth at point of inoculation within mineral-doped micromodels, organic complexed K in fungal hyphae in micromodels without minerals, natural kaolinite powder, and in a controlled etched mineral-doped micromodel not used for fungal growth. The optical microscopy image next to the organic complexed K spectra shows the fungal hyphae from where XRF maps and XANES spectra of hyphae were generated. Since this represents the first XANES spectral analysis of organic-bound K, we utilized mineral standards (natural kaolinite) and organic K spectra (from fungal inoculation point, C1 in Fig. 1a) to model our XANES data using the linear combination fitting shown in Fig. S8. Download FIG S6, JPG file, 0.9 MB. https://doi.org/10.1128/AuthorWarrantyLicense.v1 This is a work of the U.S. Government and is not subject to copyright protection in the United States. Foreign copyrights may apply.

Furthermore, hyphal trails that specifically tracked with the location of mineral grains rich in K were revealed by imaging the micromodel surface after fungal biomass was removed (see Fig. S8). This imaging was made possible because areas where the hyphae attached to the surface of the micromodel were enriched in carbon and directly observable by scanning electron microscopy (SEM) imaging. These images suggest the potential that fungi can sense and extract mineral-derived nutrients based on their requirement of a specific nutrient, K in this case. We did not detect formation of previously reported microtunnels, cavities, or micropores on the mineral grain surfaces, as observed during direct mineral weathering by ectomycorrhizal fungi (4, 33). Together, our results showing transport of K and Na through hyphae, K speciation in micromodel minerals after fungal growth, and the absence of microtunnels on micromodel mineral grains suggest that the mineral-derived nutrient extraction by Fusarium hyphae is via an indirect weathering mechanism.

10.1128/msystems.00913-22.8 FIG S8 Fungal hyphae track K-containing mineral grains on the micromodel surface. Scanning electron microscopy coupled with energy dispersive X-ray analysis of the micromodel surface after removing fungal biomass shows fungal hyphal tracks on mineral-doped micromodel surface. (a) Abundance of hyphal tracks, where Al in kaolinite is shown in yellow; (b) closer look at a hyphal track over a K-rich grain. Hyphal growth was more abundant over K-rich mineral grains, as shown in panel b. Download FIG S8, JPG file, 0.2 MB. https://doi.org/10.1128/AuthorWarrantyLicense.v1 This is a work of the U.S. Government and is not subject to copyright protection in the United States. Foreign copyrights may apply.

### P-type ATPases and transmembrane transporter proteins enable K transport from minerals into fungal biomass.

Indirect mineral weathering occurs through fungal secretion of different organic acids that are used to dissociate and uptake mineral-bound nutrients (4, 34, 35). As such, we hypothesized that Fusarium sp. DS 682 employed specific fungal transporter proteins that facilitate passage of organic acids to increase assimilation of mineral-derived nutrients into the nutrient-limited micromodel environment. We found that the fungal biomass growth within the microchannel was not sufficient for proteomics analysis. Therefore, we performed our proteomics investigation on fungal biomass on agar plates to demonstrate that this fungal strain expressed proteins in response to presence or absence of soil minerals. We expected to see fungal transporter proteins expressed in greater abundance in the presence of minerals. To test this hypothesis, Fusarium sp. DS 682 was grown in direct contact with a thin layer of natural kaolinite minerals on the surface of PDA to collect biomass for proteomics.

**FIG 3:**
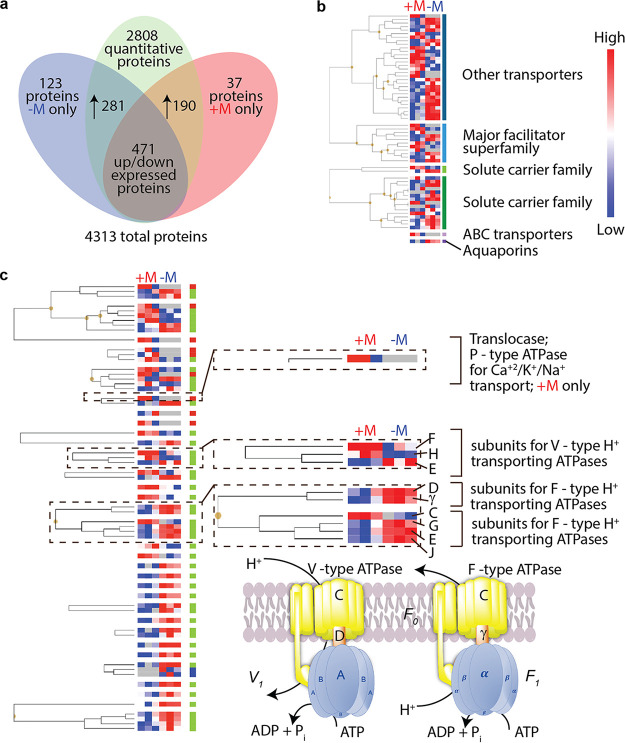
Differentially expressed fungal proteins regulate mineral substrate transport processes. (a) Venn diagram showing the number of proteins detected and enriched in fungi grown in the presence (+M) or absence (–M) of minerals. (b) Hierarchical clustering of different transporter superfamilies observed in the presence (+M) or absence (–M) of minerals. (c) Detailed hierarchical clustering of different transporter types observe in the presence (+M) or absence (–M) of minerals. KEGG pathway enrichment analysis demonstrates the upregulation of different subunits of membrane ATPases in both treatment groups (+M and –M) related to the oxidative phosphorylation pathway (see Fig. S10). The P-type ATPase for Ca^+^/K^+^/Na^+^ transport was observed in the +M treatment group alone.

**FIG 4:**
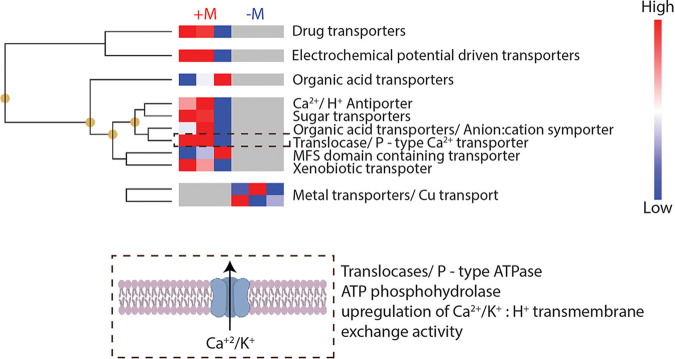
Unique transporter protein expression drives mineral transport in fungi. Hierarchical clustering of different transporter proteins expressed in the presence of minerals (+M) is evident in treatment group alone (top). These transporter proteins are not enriched in the –M treatment group. The P-type ATPase, part of the oxidative phosphorylation pathway demonstrated in detail in Fig. 3c, was expressed in the +M treatment group alone. Several organic acid transporters commonly associated with indirect mineral degradation process were also observed only in the +M treatment group. Metal transporters were enriched in the –M treatment group, as shown by our analysis. Expression of organic acid transporters in the +M treatment group alone suggests that Fusarium sp. DS 682 could use organic acids to extract nutrients in the presence of minerals.

Fungal growth was much faster in plates with minerals than without minerals (see Fig. S9a in the supplemental material). Proteins were extracted and identified by mass spectrometry, resulting in a total of 4,313 fungal proteins detected (Fig. 3a; see also Fig. S10b), where several families of transporters were expressed under both conditions, with minerals (+M) and without minerals (–M). These proteins included the major facilitator superfamily transporters, the solute carrier transporter, ATP-binding cassette-type transporters, and aquaporins (Fig. 3b). However, specific transporter proteins were enriched under both conditions. We observed 34 transport proteins significantly enriched under –M condition, while 15 transport proteins were significantly enriched under +M condition. Expression of transporter proteins under both conditions is expected since fungi will express transporters proteins for extraction of nutrients from PDA in general. Among these transport proteins, we also observed several membrane ATPases that are essential for transmembrane transport of nutrients expressed under both conditions. Specifically, several key subunits of the oxidative phosphorylation pathway, such as vacuolar membrane V-type ATPases and mitochondrial membrane F-type ATPases were detected under both conditions (Fig. 3c; see also Fig. S10). Further, we observed a P-type ATPase in the oxidative phosphorylation pathway for transmembrane transport enriched in the +M condition alone (Fig. 3c and 4). This matches with previous observations of differential expression of the oxidative phosphorylation pathway in response to mineral weathering (12). P-type ATPases in fungi are associated with increased transport of K into fungal hyphae (36, 37). Therefore, we attribute the unique expression of the P-type ATPase in the +M treatment group to increased transport of K into the fungal hyphae, as observed in our imaging data (Fig. 2a).

10.1128/msystems.00913-22.9 FIG S9 The presence of minerals induces increased fungal growth over time and growth differences reflected in the fungal proteome. (a) Fungal biomass grown on PDA with (+ minerals) or without (– minerals) minerals for 7 days. Faster growth of fungi was observed in the presence of minerals. Since proteomics analysis required a greater amount of fungal biomass than can be harvested from the microfluidic channels of the micromodels, we used the fungal biomass from agar plates amended with or without natural kaolinite. Fungal biomass for proteomics analysis was collected from agar plates, as shown here. (b) Volcano plot from ANOVA of proteomics data demonstrate the overall log fold change in protein expression in +M versus –M treatment. Statistically significant protein groups (*P* ≤ 0.05) are colored red, and increases and decreases in protein abundances are indicated as “↑+M” and “↓+M,” respectively. The specific numbers of protein expression changes from the analysis of Fusarium DS 682’s proteome in each treatment group are shown in Fig. 3a. Download FIG S9, JPG file, 0.8 MB. https://doi.org/10.1128/AuthorWarrantyLicense.v1 This is a work of the U.S. Government and is not subject to copyright protection in the United States. Foreign copyrights may apply.

10.1128/msystems.00913-22.10 FIG S10 Several metabolic transport pathways are shifted under +M and –M growth conditions. Pathways highlighted in red are unique to +M, those in blue are unique to –M, and those in green are proteins associated with shared active KEGG metabolic pathways. Using KEGG enrichment analysis, we show that ATPases specific to oxidative phosphorylation pathways are measurable in fungal growth in both the presence or absence of minerals. However, there are specific ATPases, as shown in Fig. 3c and 4, that are enriched in fungal growth only in the presence of minerals that are indicative of increased transport of mineral nutrients. A more resolved KEGG map, that is navigable, can be found in the iPath_3_ map here: Fusarium sp. DS 682 Fungal Mineral Metabolic Pathway Module Transport. Download FIG S10, PDF file, 1.9 MB. https://doi.org/10.1128/AuthorWarrantyLicense.v1 This is a work of the U.S. Government and is not subject to copyright protection in the United States. Foreign copyrights may apply.

In addition to the unique expression of P-type ATPase for Na and K transport described above, we observed nine unique transmembrane transporter proteins, such as a drug transporter (K03446), electrochemical potential driven transporters (K16261 and K07300), a sugar transporter (K08145), and a xenobiotic transporter (K05658) only in the presence of minerals (+M). Moreover, several fungal proteins associated with organic acid transport (K23630 and K03448) were enriched only in the +M treatment (Fig. 4).

## DISCUSSION

Previous studies on microbial induced mineral weathering have focused on investigating microbial metabolites and secondary minerals that are products of mineral weathering in the bulk soil (38–41). However, only a few studies have attempted to decipher weathering mechanisms by spatially exploring the fungus-mineral interface (42–44). This is in part due to the spatial heterogeneity of soil and the complexity of mineral weathering processes, which present challenges and necessitate innovative sampling and experimental platforms to study biotic-driven mineral weathering. Here, we developed a novel mineral-doped soil micromodel platform that emulates the chemical and physical complexity of soil, by embedding solid-phase minerals directly into the polymer matrix to simulate the spatially distinct hot spots of the natural soil environment that drive microbial processes underground. This platform enabled us to visualize Fusarium sp. DS 682 thigmotropic growth response and mineral dependent increase in hyphal density. We also observed increased K and Na transport and distribution within fungal hyphal networks in the presence of minerals (Fig. 2a). The extraction and uptake of K from minerals to hyphae could be due to the water stress induced within the microchannel that disconnected the PDA plugs and prevented active metabolite and carbon diffusion. However, we are not sure why there is increased uptake of Na. K is an essential macronutrient required for fungal growth, while Na uptake by fungi could have occurred simultaneously or as a substitute for K (45). Proteomics analysis of Fusarium sp. strain DS 682 biomass demonstrated expression of several different transporters (Fig. 3 and 4), perhaps to fulfill nutritional needs during fungal growth. Therefore, cation uptake captured in this work could occur through nonspecific mechanisms through cell membrane transporters. Further investigation is required to understand increased uptake of Na and K by hyphae. For example, fungal uptake of K greatly enhances hyphal growth, where hyphal uptake of K occurs through P-type ATPases (36, 37), as was found in our proteomics analysis of fungal biomass. Here, we observed fungal hyphal bridging between two carbon-rich nutrient plugs (C1 and C2, Fig. 1a and d) only in the presence of minerals. Therefore, we propose that the increase in K^+^ transport promotes hyphal growth to facilitate nutrient foraging in the water- and carbon-limited conditions of the soil micromodel environment.

We also observed changes in the K chemistry as K was transported from minerals into the fungal hyphae, where two distinct types of K chemistries, inorganic K and organic K, were observed. XANES spectra of the natural kaolinite contained pre- and postedge features that are low-intensity peaks before and after the highest intensity peaks, which are spectroscopic features consistently seen in other K minerals (46) (see Fig. S6; “powdered mineral” and “etched mineral”). We observed that the organic K spectra in fungal hyphae do not contain pre- and/or postedge features (see Fig. S6; “inoculation point” and “organic complexed”). The absence of these features could be attributed to greater distance or distortion to the nearest-neighbor ions; however, more-detailed spectroscopy is required to determine the bonding environment of the K in fungal biomass. This result that differentiates *organic* and *inorganic K* spectra is consistent with spectroscopic studies of other mineral systems, where increased crystallinity caused more photoelectron scattering events that generate postedge features (47, 48). This evidence of changes to the chemistry surrounding K, demonstrates that inorganic K was assimilated by fungi from minerals, and it suggests potential mechanisms for fungal K mineralization. Our data also present the first description of K XANES of minerals and organic compounds in the context of fungal biology. Further efforts will concentrate on characterizing the transformation and chemistry of mineral K into organic K through fungal degradation of minerals.

Collectively, the multimodal chemical imaging revealed that the fungal growth in mineral-doped micromodels during nutrient and water limitation occurred through fungal weathering and uptake of mineral-derived nutrients. Moreover, we observed increased expression of unique transporter proteins and the presence of organic acid transporters in only the +M condition through fungal biomass proteomics analysis. This evidence supports our hypothesis that this fungus is degrading and assimilating mineral-derived nutrients to support enhanced growth in mineral-rich environments compared to the mineral-free control. Furthermore, the enriched expression of organic acid transporters in the +M condition in this strain of fungus, together with the absence of microtunnels or pores on mineral grains (see Fig. S8), suggests that the Fusarium sp. DS 682 likely degrades minerals through indirect weathering. Indirect mineral weathering could also be due to the short timescale investigated here, as all evidence of direct weathering in soil experiments have explored longer timescales than what we explored. Further investigation is required to confirm this observation.

The K transport into fungal hyphae, K speciation in the micromodel minerals, and the presence of organic acid transporter proteins in fungal biomass grown with mineral together suggest that Fusarium sp. DS 682 has a strong weathering effect on minerals through indirect mechanisms. Previous work on biotic mineral weathering suggests that fungi can sense different mineral types and direct resources toward mineral weathering under specific conditions, such as low-moisture and low-nutrient environments (49, 50). However, the underlying molecular mechanisms by which soil fungi extract and transport mineral-derived nutrients are largely unknown, and direct transport of nutrients to fungi from the mineral surface have not been characterized. Here, by using mineral-doped soil micromodels and advanced imaging technologies, we demonstrated fungal bridging of carbon-rich hot spots, mineral weathering, and transport of mineral-derived nutrients by a saprotrophic soil fungus at microscale-level resolution. This platform can enable future studies of different soil fungus processes related to thigmotropic growth response, mineral dependence, nutrient translocation, and beyond. Moreover, the mineral doping technology is versatile and adaptable to any microfluidic device design. Therefore, mineral doping in micromodels could facilitate future investigation into plant and microbial mineral weathering to generate a comprehensive understanding of biotic weathering mechanisms of soil minerals and inorganic nutrient cycling.

## MATERIALS AND METHODS

### X-ray diffraction.

X-ray diffraction was performed using a Rigaku Rapid II microbeam diffractometer (Rigaku Corporation, Tokyo, Japan) equipped with a rotating Cr anode (λ = 2.2910 Å) and a large two-dimensional image plate detector. The patterns were collected in transmission mode from an ~0.5-mm slice of the PDMS/kaolinite composite and a small aggregate of kaolinite mounted in a glass fiber. A 300-μm-diameter incident beam was used, and the image plate intensities were converted to powder traces using Rigaku 2DP software. Quantitation of the mineral composition was carried out by whole-pattern (Rietveld) fitting with TOPAS v6 (Bruker AXS) and crystal structures published in the Inorganic Crystal Structure Database (Fachinformationszentrum, Karlsruhe, Germany). This method provides the relative fractions of crystalline minerals observed. The natural kaolinite contains relative fractions of kaolinite (85%), K-feldspar (7%), quartz (4%), and mica (4%). The analysis of these fractions is scaled to 100%, ignoring any amorphous materials.

### SEM and EDX analysis.

Sections were cut out from PDMS micromodels for SEM analysis to fit into the SEM sample holder. For analysis of the PDMS micromodel surface after fungal growth (see Fig. S9), the fungal biomass was removed by exposing the PDMS surface with fungi to 2% (wt/vol) *N*-dodecyl-β-d-maltoside in water. After which, sections were cut from the PDMS micromodel to fit the SEM sample holder. All micromodel sections were mounted into aluminum stubs. To reduce sample charging, the sections were coated with ~15-nm carbon using a thermal evaporation method (108C Auto Carbon Coater; Ted Pella, Inc.) and secured with Cu sticky tape on the SEM stage. SEM analyses were conducted with a FEI Helios NanoLab 600i field emission electron microscope. Images were collected with Everhart-Thornley secondary electron detector (ETD) in a field-free mode and at an acceleration voltage of 3 to 5 kV, a current of 0.086 to 0.17 nA, and an ~4-mm working distance. Backscattering images were also collected, applying low (3 kV) and high (10 kV) settings to identify PMDS locations (hyphal tracks, Fig. S9) occupied by fungal hyphae and correlate the hyphal tracks with the positions of mineral grains that were incorporated within the PDMS.

The chemical composition of grains in the PDMS and elemental distribution maps, which illustrate hyphae paths and distribution of mineral grains, were collected with an energy dispersive X-ray detector (EDX). The SEM was equipped with an EDX X-Max 80-mm^2^ Silicon Drift Detector (Oxford Instruments, Abington, UK) capable of detecting lighter elements. EDX analyses were performed at 3 kV to visualize C-elemental distribution and at 10 kV to collect chemical data on K, Ca, Mg, Si, and Al. Oxford INCA software was used to collect compositional maps (acquisition time, >300 s) and point spectrum analyses (acquisition time, ~80 s).

### Fungal strain extraction and isolation.

The soil fungus Fusarium sp. DS 682 was used for the experiments in this study. This strain was isolated from Kansas Prairie Biological Station (KNZ), as previously described (29). Fungal spores were obtained from the fungus to use as inoculum for the experiments. To extract spores, 10 mL of 10% sterile glycerol in deionized (DI) water was added to a hyphal mat of Fusarium sp. DS 682 grown on PDA plates for 6 days. The plate was gently tipped in all directions to ensure even distribution of glycerol. The liquid from the plate was removed using a sterile pipette and transferred through a 40-μm filter cap into a 50-mL Falcon tube. Then, 1-mL aliquots of the spore suspension were transferred into 1.5-mL safe-lock Eppendorf tubes and stored at −80°C until use.

### Fungal strain culture conditions.

Next, 100-μL portions of Fusarium sp. DS 682 spore suspension were added to M9 PDA plates, followed by incubation at 28°C for 2 weeks. M9 PDA medium was prepared by adding micronutrients (see recipe below), 24 g/L potato dextrose (BD Biosciences, San Jose, CA), and 2% granulated agar (BD Biosciences) to M9 salts (MP Biomedicals, Irvine, CA) in Millipore water. The micronutrients consisted of 0.145 mM ammonium molybdate (Thermo Fisher Scientific), 2 mM boric acid (Sigma-Aldrich, St. Louis, MO), 0.151 mM cobalt chloride (Sigma-Aldrich), 0.048 mM cupric sulfate (Sigma-Aldrich), 0.404 mM manganese chloride (Sigma-Aldrich), and 0.048 mM zinc sulfate (Thermo Fisher Scientific). These micronutrients were added after autoclaving the M9 PDA solution, when the solution was ~55°C. After pouring the M9 PDA into petri dishes, the solidified agar plates were wrapped in Parafilm and stored at 4°C for future use. M9 PDA plates were primarily used to maintain the fungal cultures and not in the micromodel experiments described in this paper. The fungal experiments in the micromodel and for proteomics analysis were conducted using PDA plugs and PDA plates, respectively, as described in detail below.

### Micromodel Si master fabrication.

The soil micromodels were generated using the SU8 photolithography and PDMS soft lithography processes (51). Briefly, a chrome mask with the micromodel pattern (Fig. 1) was generated in-house using a mask printer (Intelligent Micro Patterning, LLC, St. Petersburg, FL). The silicon masters were prepared by spin coating SU8 25 (Kayaku Advanced Materials, Inc., Westborough, MA) at subsequent spin speeds of 600 and 2,000 rpm for 10 and 30 s, respectively, to create a thin SU8 photoresist film using a WS-400B-6NPP/LITE spin coater (Laurell Technologies Corp., North Wales, PA). The silicon wafer with the SU8 layer was heated at 65°C and 95°C for 3 and 7 min for prebaking, respectively, before exposure to 350-nm wavelength of light at 1 W/cm^2^ for 40 s using a mask aligner (NXQ-4006 Contact Photomask Aligner; Neutronix Quintel, Morgan Hill, CA) to cross-link the photoresist. After UV exposure, the Si wafer was baked at 65°C and 95 ° C for 1 and 4 min, respectively, for postexposure bake and developed using SU8 developer (Kayaku Advanced Materials). Finally, the Si wafer with the micromodel image was hard baked at 180°C for 20 min. The micromodel contained pore throats of 12 μm with pillars, representing soil aggregates of 300 μm placed 150 μm apart.

### Micromodel fabrication.

Polydimethylsiloxane (PDMS; Dow Corning, Midland, MI) soil micromodels were created by mixing the PDMS base and curing polymers at a 10:1 ratio and then poured over the Si micromodel channel master. The PDMS was then cured at 70°C for 2 h. To create PDMS micromodels that contained minerals, the same base and curing polymer mixing ratio was used and natural kaolinite powder (Sigma-Aldrich) was added at 10% (wt/vol) to the liquid PDMS and mixed well with a spatula. The PDMS mineral mixture was poured over the SU8 negative channels, and the mineral grains were allowed to sediment overnight (~15 h) and cured at 70°C for 2 h. For reversible binding of the PDMS soil micromodels to glass coverslips (24 mm × 60 mm; Chemglass Lifesciences, Vineland, NJ), the coverslips were coated with a layer of uncured PDMS (base/curing polymer ratio of 20:1) by spin coating at subsequent spin steps of 600 and 3,000 rpm for 10 and 30 s, respectively. They were then cured by placing the coated coverslip on a hot plate for 10 min at 110°C, creating a sticky PDMS substrate. Creating inoculation port covers for the devices followed the same 20:1 ratio PDMS recipe, and 20 mL of this mixture was poured into a 150-mm-diameter petri dish (VWR, Radnor, PA) and cured at 70°C for 30 min. The cured polymer was cut into squares (6 mm × 6 mm) and plasma cleaned (PX250; Nordson March, Concord, CA) before being used to seal the inoculation ports of the micromodels.

### Micromodel etching.

The micromodel surface was etched to expose minerals embedded in the PDMS matrix (see Fig. S2) using deep reactive ion etching (Plasmalab System 100; Oxford Instruments–America, Inc., Concord, MA) using a combination of sulfur hexafluoride (45 standard cm^2^/min [SCCM]) and O_2_ (5 SCCM) gases for 1 min. The etching process had no measurable effect on the mineral chemistry (see Fig. S2b). After etching, holes were punched at the two ends of the micromodel channel using a 5-mm hole punch for placing agar plugs (nutrient) and fungal biomass (starting inoculum) (C1 and C2 in Fig. 1a, respectively). The PDMS micromodels and glass coverslips coated with PDMS were cleaned by rinsing with ethanol and sterile DI water and then dried using compressed nitrogen before exposing the samples to oxygen plasma (PX250; Nordson March) for 30 s. The micromodel and PDMS-coated glass coverslips were then left undisturbed in Parafilm-sealed petri dishes on the counter for 24 h to allow diffusion of methyl groups to the surface of the PDMS that prevented irreversible bonding once the PDMS micromodel and PDMS-coated glass coverslips were brought into contact. The channels were tested for leaks, and the sticky PDMS coating prevented any fungal growth outside the channel.

### XPS analysis.

XPS data shown in Fig. S2b were acquired by using a Quantera Scanning X-ray Microprobe (Physical Electronics, Inc., Chanhassen, MN). This system uses a focused monochromatic Al Kα X-ray (1,486.7 eV) source for excitation and a spherical section analyzer. The instrument has a 32-element multichannel detection system. The X-ray beam is incident normal to the sample, and the photoelectron detector is at 45° off-normal. High-energy resolution spectra were collected using a pass energy of 69.0 eV with a step size of 0.125 eV. For the Ag 3d_5/2 _line, these conditions produced a FWHM of 0.92 eV ± 0.05 eV. The binding energy scale is calibrated using ISO 15472 Ed.2 Surface Chemical Analysis–XPS–Calibration of energy scales. The Cu 2p_3/2_ feature was set at 932.62 ± 0.05 eV, and the Au 4f_7/2 _line was set at 83.96 ± 0.05 eV. The sample experienced variable degrees of charging. Low-energy electrons at ~1 eV and 20 μA and low-energy Ar^+^ ions were used to minimize this charging. Quantification was performed using Multi-Pak software, v9.1.1.7 (ULVAC-PHI, Inc.).

### Fungal inoculation and growth conditions in soil micromodels.

A disposable 1.5-mm holepunch (Integra Miltex, York, PA) was used to generate agar pieces to fill into the inoculation port (1 agar plug) and the second nutrient port (4 agar plugs). By using another 1.5-mm holepunch, we collected the fungal biomass from a fungal culture agar plate (1 agar plug with fungi), and this was inoculated via the inoculation port. The agar plugs were generated from PDA without M9 media and micronutrient addition. The ports were then sealed with ~2-mm-thick sticky PDMS inoculation port cover membranes. The micromodels with fungal biomass inoculation were incubated at 28°C for 7 days.

### Characterization of fungal growth and hyphal density analysis.

Fungal growth in the micromodels were imaged using a Nikon eclipse TE2000-E epifluorescence microscope (Nikon, Melville, NY). Mosaic bright-field images were obtained using a 4× air objective and stitched across the length and width of the microchannel to capture fungal growth in micromodels. Prior to processing, each image was cropped to the same square dimensions (6,115 × 6,115 pixels) to analyze only the area shown in Fig. S4. The structured region was not included in the image analysis, since in micromodels without minerals, fungi did not grow into the pore spaces and the structures created artifacts that convoluted the image analysis results. The cropped images were imported into ImageJ (52), and each image type was changed to an 8-bit image before further processing. Next, the image threshold was adjusted manually to create the most contrast between the fungal hyphae and the sample background. The image was then inverted, making the fungal biomass appear white against a black background, and the images were made binary, which sets a threshold at where ImageJ determines if a pixel is either black or white (collapsing the 256 intensity channels down to 2). The hyphal density was then determined from the ratio of white pixels in an image to the total image. Ten devices per condition were analyzed to obtain the average density and standard deviations per condition.

### Functionalization of PDMS surface.

The surface chemistry of the PDMS micromodels without minerals was changed to determine the effect of surface chemistry on fungal growth (see Fig. S3b). The surfaces were rendered hydrophilic or hydrophobic by vapor phase functionalization using 3-aminopropyltrimethoxy silane or chlorotrimethylsilane (Sigma-Aldrich), respectively. The PDMS micromodels were plasma cleaned for 30 s and placed in a vacuum desiccator (Cole-Parmer, Vernon Hills, IL), along with 100 μL of silane solution in an aluminum boat. The micromodels were functionalized after placement in a vacuum for 15 h, followed by baking at 70°C for 2 h.

### ToF-SIMS analysis.

The micromodels with fungal growth were disassembled by removing the PDMS membrane covers and PDMS-coated glass coverslips and then analyzed using a TOF.SIMS5 instrument (IONTOF GmbH, Münster, Germany). A 25-keV pulsed Bi_3_^+^ beam was focused to an ~5-μm diameter and scanned over a 500-μm-by-500-μm area to collect SIMS spectra and images. The current of the pulsed Bi_3_^+^ beam (10 kHz) was 0.56 pA, while the data collection time was about 328 s per spectrum with a mass resolving power between 4,000 and 7,000 across the spectra. A low-energy (10 eV) electron flood gun was used for charge compensation in all measurements. The ToF-SIMS data were analyzed using SurfaceLab software (v6.4), which was provided by the instrument manufacturer.

### Synchrotron experiments.

The micromodels with fungal growth were disassembled by removing the PDMS membrane covers and PDMS-coated glass coverslips. The micromodel surface with fungi was then exposed to 4% paraformaldehyde (PFA) vapors by placing opened micromodels with a PFA-soaked filter in a petri dish for 24 h at room temperature, which arrested fungal growth. Micromodels that contained no fungi, as well as a natural kaolinite powder, were used as controls, and these samples were not exposed to the PFA protocol. Micro-X-ray fluorescence (μ-XRF) imaging and X-ray absorption near edge structure (XANES) spectroscopy were conducted using the tender X-ray microprobe beamline 14-3 at the Stanford Synchrotron Radiation Lightsource (SSRL), SLAC National Accelerator Laboratory. Both μ-XRF imaging and XANES spectroscopy were performed around the potassium K-edge (3,608 eV) using a water-cooled double Si crystal (111) monochromator to select the incident X-ray energy with a beam size of 5 μm. Natural kaolinite powder (Sigma-Aldrich) was used to calibrate the monochromator using the position of the white line at 3,618.73 eV. The micromodels were mounted onto a 360° rotating sample wheel and placed into the He-purged sample chamber (O_2_ in the sample chamber was purged to <0.02% before analyzing the sample in order to reduce argon interference). Initially, μ-XRF images were collected at a coarse resolution (10-μm step size) above the potassium K-edge (3,625 eV) in order to capture all chemical forms of K. In addition, XANES spectra were obtained on (i) natural kaolinite powder, (ii) minerals in the micromodel without fungal growth, (iii) minerals in the micromodel with fungi growth, (iv) the fungal inoculation point, and (v) a micromodel with fungal growth but without minerals (see Fig. S6; defined as end-member spectra) in order to determine the number of different chemical forms of K present in the samples for multi-energy (ME) mapping. ME maps were collected at 3,615.65, 3,617.25, 3,618.4, 3,620.15, and 3,627 eV based on features identified in the XANES spectroscopy end members (organic K and kaolinite mineral standards). High-spatial-resolution ME maps were acquired with a 3-μm step size in order to oversample the regions of interest.

### Synchrotron data processing.

Data were processed using the MicroAnalysis toolkit (53), SixPACK, and Athena X-ray absorption spectroscopy software packages (for XANES spectroscopy) (54, 55). During data collection, principal-component analysis (PCA) was employed on the ME maps to determine unique components within the mapped region to guide the locations for further investigation with XANES spectroscopy. XANES spectra were processed using a detector dead-time correction, background subtraction of the linearized pre- and postedge values, and edge-step normalization. XANES spectra were fit using a linear combination fitting of the end-member spectra (i.e., the only known K complexes within the sample; Fig. S6 and S7). Subsequently, a cross-plot of the individual components from PCA of the processed XANES spectra from each mapped region was used to identify the spectra that are the most dissimilar to one another (i.e., most different K chemistries). A least-squares fitting of these spectra to the ME maps was generated to a second set of spatial abundance maps of the same region in order to further show the distinct K chemistries (56–58). Prior to this experiment the differences between XANES spectra of inorganic versus organic complexed K was unknown. In these experiments, K complexed inorganically (in minerals) and organically (in fungal hyphae) had very similar white line positions, but with different characteristics in the pre- and postedge values. This meant that each ME map appears very similar, and a least-squares XANES fitting was required to tease apart the locations of organic complexed K from mineral-bound K.

### Fungal proteomics.

The fungal samples for proteomics analysis were extracted from fungal biomass grown on PDA agar without M9 and micronutrients. To expose the fungus to minerals, 100 μL of 50% (wt/vol) natural kaolinite solution in water was autoclaved and spread over the agar surface by using a disposable L shaped spreader (Thermo Fisher Scientific, Waltham, MA). The solution was dried for 1 h to create a thin mineral film on the agar surface. Three plates each with (+ mineral) and without mineral (− mineral) condition were inoculated with fungal biomass from 14 days of growth on PDA using a 1.5-mm holepunch. The plates were incubated at 28°C for 15 days before extracting hyphae (500 mg) from each plate. The proteins were extracted using the MPLEx protocol, as previously described (59), and further details regarding sample preparation and data analysis are provided below.

### Proteomics sample preparation.

The hyphal biomass was extracted in microcentrifuge tubes with beads (Omni International, Kennesaw, GA) and 50 mM NH_4_HCO_3_ (pH 8) and then lysed using a Bead Ruptor Elite (Omni International) tissue lyser by homogenizing the sample twice at 6 rpm for 45 s. The microcentrifuge tube with the cell lysate was placed over a 15-mL falcon tube after a hole was poked in the microcentrifuge tube using a 26-gauge needle, where the lysate was then extracted by centrifugation at 4,500 × *g* for 5 min, and the microcentrifuge tube with beads was washed using ammonium bicarbonate solution and centrifuged again. The lysates from the two wash cycles were combined.

A 2:1 solution of CH_3_Cl:CH_3_OH was added at five times the volume of the cell lysate, vortexed for 30 s, kept on ice for 5 min, and vortexed again for 30 s. The solution was centrifuged at 10,000 × *g* for 10 min at 4°C, and the protein interlayer was collected and dissolved in 8 M urea. A BCA protein assay (60) was performed to determine the amount of protein in each sample before further analysis using a global digestion protocol. The protein interlayer solution in urea was then incubated at 60°C after adding dithiothreitol (Sigma-Aldrich) at a 5 mM concentration for 30 min. The sample was diluted 10-fold in 50 mM NH_4_HCO_3_, and then CaCl_2_ was added to a concentration of 1 mM. Protein digestion was performed by incubation in trypsin (1 μg trypsin/50 μg protein; Thermo Fisher Scientific) for 3 h. The samples were desalted using 30-mg/mL C_18_ polymeric reversed-phase column (Strata-X 33u; Phenomenex, Torrance, CA) and analyzed by liquid chromatography-mass spectrometry (LC-MS).

### LC-MS analysis of protein digests.

A nanoACQUITY ultraperformance liquid chromatography (LC) with a 2DLC system (Waters, Milford, MA) was used for separation of protein digests. Buffer A (0.1% formic acid in water) and buffer B (0.1% formic acid in acetonitrile) were used as mobile phases for a gradient separation of 180 min. First, 5-μL protein digests were automatically loaded onto a C_18_ reversed-phase column prepared in-house (70 cm by 70 μm [inner diameter], with 3-μm Jupiter C_18_ particle size, room temperature) with 100% buffer A at 5 μL/min. The eluted peptides from the C_18_ column were analyzed by using a Q-Exactive Plus Orbitrap MS (Thermo Scientific, San Jose, CA) for high-resolution MS and high-energy collision-induced dissociation tandem MS by electrospray ionization. Samples were analyzed using a 180-min LC-MS/MS method, and data acquisition commenced 15 min after sample injection. Spectra were collected between 375 to 1,800 *m/z* at a mass resolution of 180,000 (at *m/z* 200), following by a maximum ion trap time of 180 ms. Peptides were fragmented using a high-energy collision energy level of 32% and a dynamic exclusion time of 30 s for discriminating against previously analyzed ions.

### Proteogenomic sequence database construction for proteomics analysis.

Fusarium sp. DS 682 required additional protein sequence annotation support using constructive proteogenomic database search methods (61–63). Fusarium sp. DS 682 was recently sequenced (29, 64, 65) and annotated using the recently released genome annotation pipeline Funannotate (v1.7.0; Palmer and Stajich, GitHub [https://github.com/nextgenusfs/funannotate/tree/v1.7.0]), which is specific for fungal and higher-order eukaryotic genomic annotation. Functional gene mapping for protein predictions of Fusarium sp. DS 682 resulted in 19,949 predicted gene product protein sequences, for which 44% of the proteome sequences contained a predicted KEGG ortholog (KO) protein annotation assignment (8,849 total genome KO IDs mapped), leaving 56% of the proteome unannotated. To supplement proteome sequence annotation deficiencies, additional searches were performed using BLASTP to incorporate additional mapped reference protein sequence fragments from subselected proteomes of corresponding genomes from the closest model orthologs. The model orthologs used were as follows: Fusarium graminearum (GCA_900044135.1; mash [66] distance = 0.21 [k = 17, s = 100,000, as suggested previously] [67]; 7,548 reference protein annotation transfers from 14,162 proteins), Fusarium pseudograminearum (GCA_000303195.2, 36.5 Mb; mash distance = 0.21; 7,377 reference protein annotation transfers from 12,448 proteins), and Fusarium oxysporum *f.* sp. *lycopercisi* (GCA_003315725.1, 36.5 Mb; mash distance = 0.14; 11,630 reference protein annotation transfers from 16,646 proteins). These species were selected because they represented a functionally diverse set of phylogenetic clades and mimic a similar geographical host/substrate grassland soil environment (68–70). Ortholog supplementation permitted annotation of an additional ~16% of the Fusarium sp. DS 682 proteome.

### Proteogenomic data analysis.

Label-free Fusarium sp. DS 682 raw sequence data files were processed using MaxQuant (71, 72) (v1.6.7.0) for feature detection, comparative peptide sequence database searches, and protein quantification for subsequent downstream proteomic analysis for biological interpretation. Proteomic sample collection data set groups for Fusarium sp. DS 682 consisted of two fungal growth treatments, with (+M) or without (−M) minerals, where each treatment group contained three biological replicates. Raw fungal data set files were grouped by assigned treatment group and analyzed using a standard LC-MS run type. N-terminal protein acetylation and methionine oxidation were selectively applied as a variable modification for group-specific parameters, allowing a maximum of three modification per peptide. Peptides used for parent sequence matches required a minimal of one peptide observation per protein, per organism collection file, and a minimum peptide length of seven amino acids. Sequence digest parameters were restricted to an allowed maximum of two missed tryptic cleavages with an MS/MS tolerance of 20 ppm. For increased peptide/protein identification, match between runs (MBR) employed with a 20-min alignment window (0.7-min time match window) using a peptide/protein (unique + razor matches) match false discovery rate (FDR) cutoff of ≤ 0.01 (1% FDR).

Peptide fragments were quantitatively searched against a constructed proteogenomic sequence database using a suite of organism protein collection files for F. graminearum (GIBZE)—Fusarium_graminearum_PH-1_SPROT_TrEMBL_2019-10-16.fasta (downloaded on 16 October 2019 containing 14,160 protein entries), F. pseudograminearum (FUSPC)—Fusarium_pseudograminearum_CS3096_SPROT_TrEMBL_2019-12-13.fasta (downloaded on 16 October 2019 containing 12,448 protein entries), F. oxysporum (FUSOX) top strains—F_oxysporum_SPROT_TrEMBL_2019-11-21.fasta (downloaded on 16 October 2019 containing 70,763 protein entries), and the resulting predicted protein sequence file for Fusarium sp. DS 682 (SF-1-001.genemark_KOfam_V2_May2020_new.fasta). All other parameters applied not listed here were ran using MaxQuant software defaults.

### Statistical preprocessing of proteomics data.

Statistical preprocessing was performed on resulting data set MaxQuant output file (“proteinGroups.txt”) containing iBAQ absolute protein abundance data for both mineral treatment groups and the corresponding biological sample replicates (*n* = 3). Data sets were first filtered for removal of all potential contaminants and reverse hits. The following criteria was used to further filter reference sequence database match quality using proteogenomic search methods prior to statistical analysis. Data set peptide/protein sequence matches were filtered out from the data for either protein groups where (i) the majority protein identifier was comprised of only ortholog sequence file matches or (ii) the majority protein identifier listed had multiple proteins associated with the same redundant peptide fragment matched within a given reference organism collection file. Finally, for assessing reproducibility, mineral treatment groups with too few replicate observations to conduct a quantitative or qualitative statistical comparison were removed (i.e., at least two observed abundance values per mineral treatment group or at least three observed abundance values in one group). The remaining 2,808 proteins passing match-quality filter criteria for subsequent statistical analysis were log_2_ transformed, and all nonobserved values were assigned a value of NA. Filtering did not change the abundance profiles distributions.

### Statistical analysis of proteomics data.

SPANS software (73) was utilized for evaluating potential data normalization strategies resulting in data normalized via median centering. Differential analysis of log_2_-transformed relative abundance expression data was applied to assess quantitative (analysis of variance [ANOVA]) and qualitative (g-test) statistical significance. A one-way ANOVA (with a *P*-value cutoff of 0.05) test was run for each fungal mineral treatment group for comparing mean abundances (log_2_) across biological replicates. A g-test was run for evaluating qualitative differences in presence/absence patterns of significantly expressed proteins identified in either treatment group by using a null hypothesis that presence/absence patterns are not related to a biological group. The resulting ANOVA test flags (quantitative change) and g-test flags (qualitative change) indicating the direction and range of significant change in protein expression values (0, not significantly expressed; 1, significantly expressed in the +M treatment group; and −1, significantly expressed in the −M treatment group) were filtered (cutoff of ±1, ANOVA/g-test flags; ≤ 0.05, ANOVA *P* values) for representing the topmost proteins observed to be significantly changing in expression or uniquely observed by the presence/absence in one mineral treatment group over the other. A total of 160 proteins were uniquely observed by g-test (presence/absence) in only one mineral protein group. Unique protein observations are defined as being observed in only one mineral treatment group and having abundance values for 2/3 across proteomic sample replicates within a given treatment group. To explore sequence data match-quality statistics supplementing this proteogenomic method analysis, see our publication data at 10.25584/KSOmicsFspDS682/1766303 which is available for download.

### Functional metabolic pathway analysis.

Fusarium sp. DS 682 predicted protein KEGG Ortholog (KO) assignments from resulting statistical analyses for quantitative and qualitative significance, for both mineral groups, were mapped to metabolic pathways using KO reference database resource KEGG Mapper (74, 75). This permitted linking genomes to pathways by functional orthologs. Statistically significant Fusarium sp. DS 682 proteins not assigned to a predicted KO ID were omitted from KEGG Mapper entity list. However, if an unassigned predicted Fusarium sp. DS 682 protein entity was matched to a corresponding cross-reference ortholog KO assignment using the MaxQuant database search methods (passing quality match filter criteria above), then the ortholog KO was adopted by default, or an available KO number was obtained using protein sequence BLASTP search results if applicable. KEGG Mapper metabolic outputs linked proteogenomic sequence predictions to functional ortholog annotations for metabolic pathway maps (ortholog molecular interactions, reactions, and relations), BRITE hierarchies (ortholog functional hierarchies of biological pathway or reaction entities), and KEGG modules (complete ortholog modular units of a conserved function, such as conserved reaction transport complexes). Of 631 statistically significant proteins observed, 495 matching a nonredundant predicted KO assignment (iPath_3_ map Fusarium sp. DS 682 Fungal Mineral KO Metabolic Pathways) were mapped to 300 metabolic pathways, 41 BRITE hierarchies, and 105 KEGG modules. Pathway analysis revealed specific metabolic shifts in fungal Central Energy Metabolism in response to mineral addition (iPath_3_ map Fusarium sp. DS 682 Fungal Mineral KO Metabolic Pathway Modules). For mapping predicted transporter protein activity for both treatments, protein KO entities with an observed increase in quantitative abundance (ANOVA, *P* value cutoff of 0.05) or unique presence/absence (by g-test = +1) were further evaluated. Fusarium sp. DS 682 fungal transporter proteins were mapped to 54 metabolic pathway maps, 15 BRITE hierarchies, and 5 KEGG reaction modules, revealing a significant shift in central metabolic energy transfer storage and translocation mechanisms (iPath_3_ map Fusarium sp. DS 682 Fungal Mineral Metabolic Pathway Module Transport). A total of 60 predicted cross-ortholog protein transporters were identified for Fusarium sp. DS 682 identified belonging to five major active membrane trafficking involved transporter families (Fig. 3b; see also Fig. S10).

### Data availability.

High-throughput omics project data reported here, including subsequent downstream process method metadata, have been uploaded to the Pacific Northwest National Laboratory DataHub: Scientific Data Repository (https://data.pnnl.gov/) as a comprehensive digital dataset package accessible for download from the Phenotypic Response of the Soil Microbiome to Environmental Perturbations Project homepage (https://doi.org/10.25584/PRJ.SFA/1780765), under the digital object identifier https://doi.org/10.25584/KSOmicsFspDS682/1766303 for dataset “KS4A-Omics1.0_FspDS682 (Fungal Monoisolate Multi-Omics Proteome, KS)”. Data package contents reported here are the first version (1.0) and contain both primary and secondary mass spectrometry, mass spectroscopy, microscopy, and optimal imaging capability data. Data release contents are structured to meet compliance with domain community initiative standards supporting FAIR data principles, and guidelines outlined by the Genomic Science Program Data Sharing Policy (https://genomicscience.energy.gov), for reuse at FAIRsharing (https://fairsharing.org/) digital object identifier https://doi.org/10.25504/FAIRsharing.19ne3m. Primary raw mass spectrometry liquid-chromatography data and corresponding instrument parameter metadata have been deposited at the Mass Spectrometry Interactive Virtual Environment (MassIVE) public domain community repository, supporting the free exchange of mass spectrometry data for reuse, and are accessible for FTP download under the digital object identifier https://doi.org/10.25345/C50V4N (MassIVE Accession: MSV000087221) [dataset license: CC0 1.0 Universal (CC0 1.0)]. Proteogenomics statistical software data processing and workflow analysis, including code for resulting interactive Trelliscope plots, can be found at the GitHub repository https://github.com/lmbramer/Fusarium-sp.-DS-682-Proteogenomics.
