# Supplementary figures and images for: A Mineral-Doped Micromodel Platform Demonstrates Fungal Bridging of Carbon Hot Spots and Hyphal Transport of Mineral-Derived Nutrients
Source: mSystems. 2022 Nov 17;7(6):e00913-22. doi: 10.1128/msystems.00913-22 (PMC9765027; doi:10.1128/msystems.00913-22)

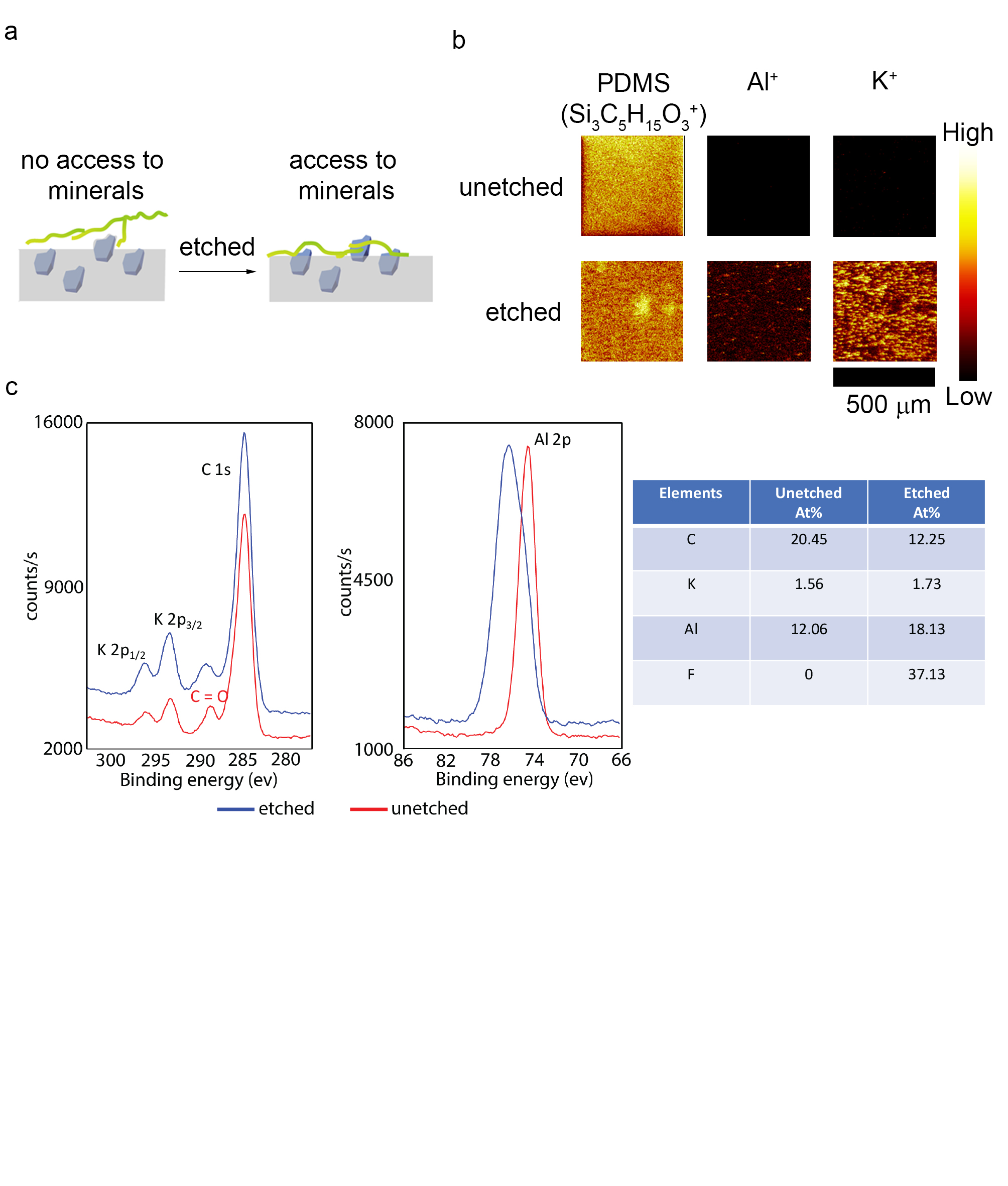

Supplement: FIG S2 [file msystems.00913-22-s0002.jpg]

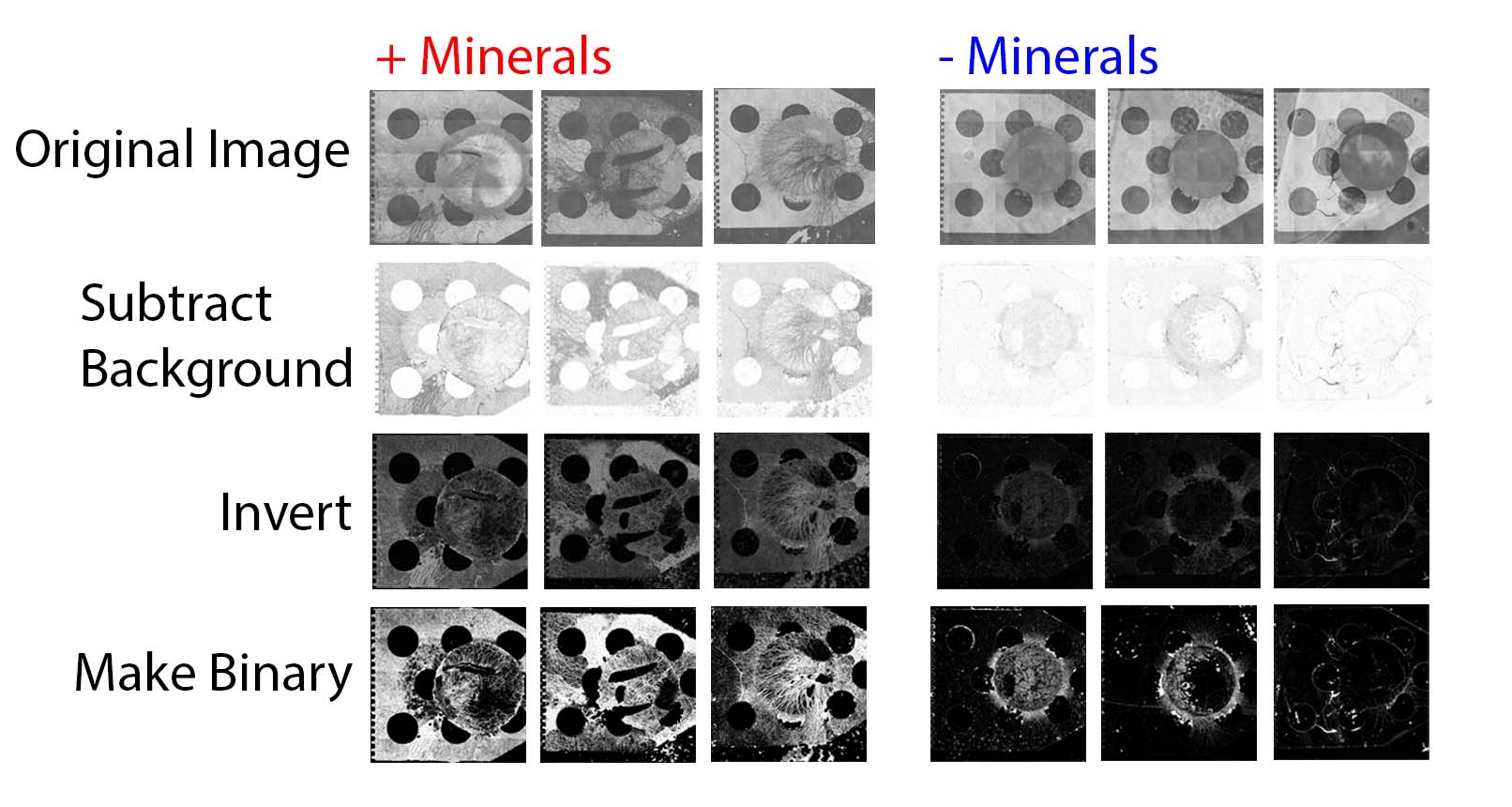

Supplement: FIG S4 [file msystems.00913-22-s0004.jpg]

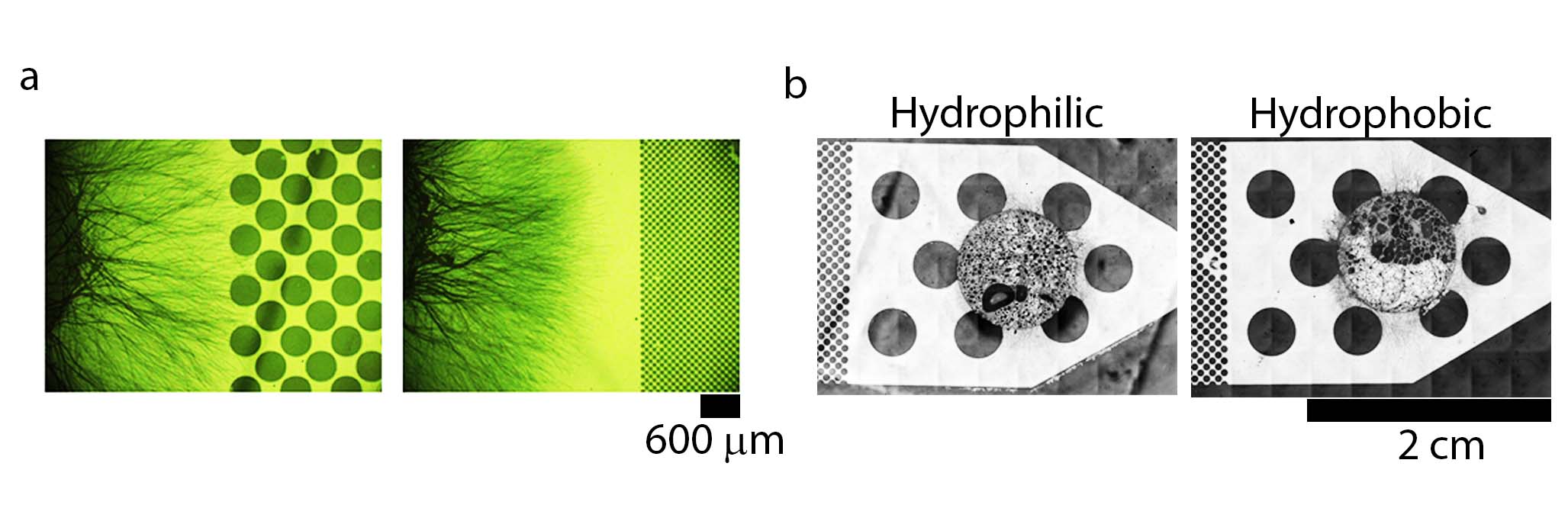

Supplement: FIG S3 [file msystems.00913-22-s0003.jpg]

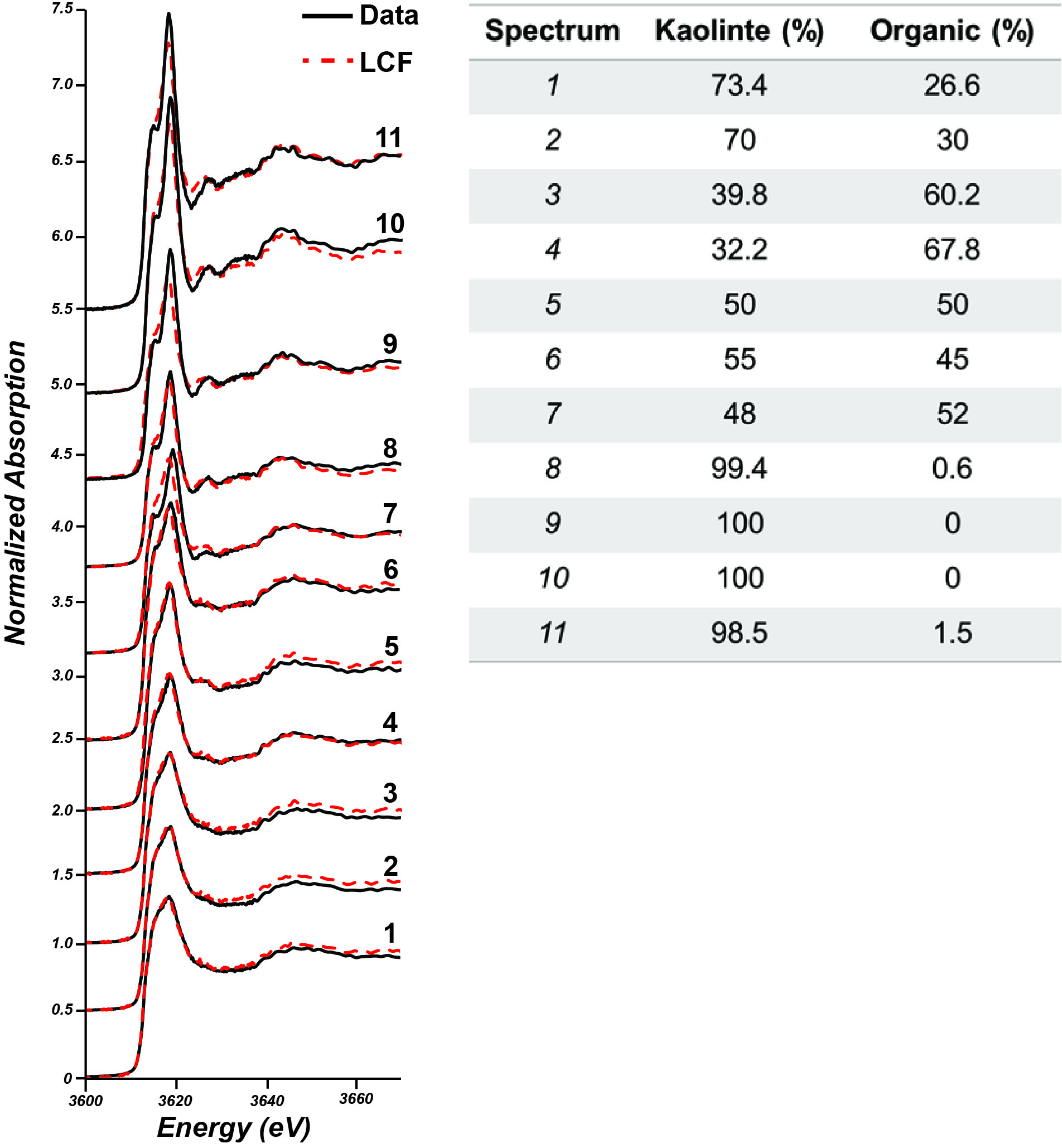

Supplement: FIG S7 [file msystems.00913-22-s0007.jpg]

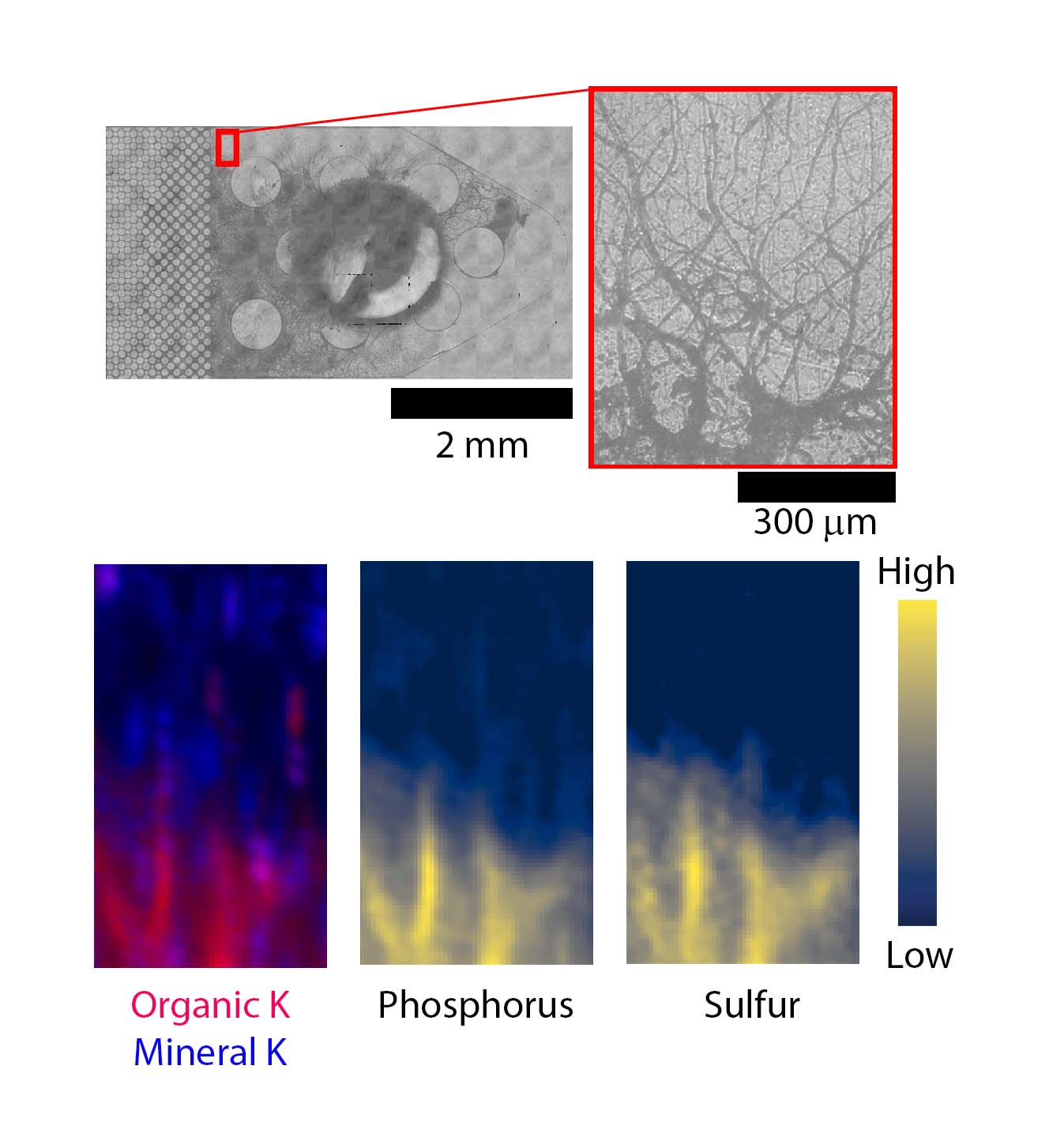

Supplement: FIG S5 [file msystems.00913-22-s0005.jpg]

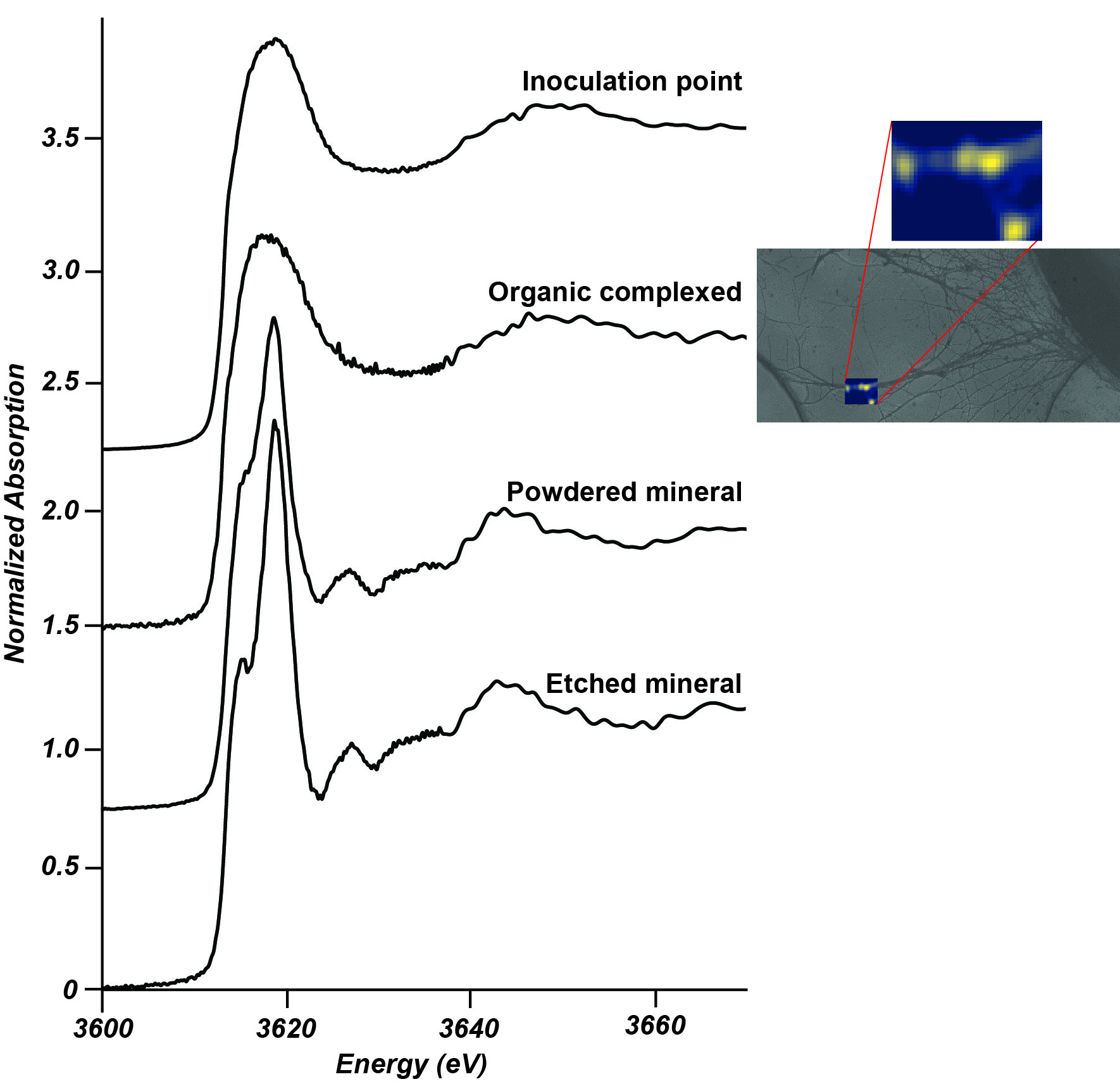

Supplement: FIG S6 [file msystems.00913-22-s0006.jpg]

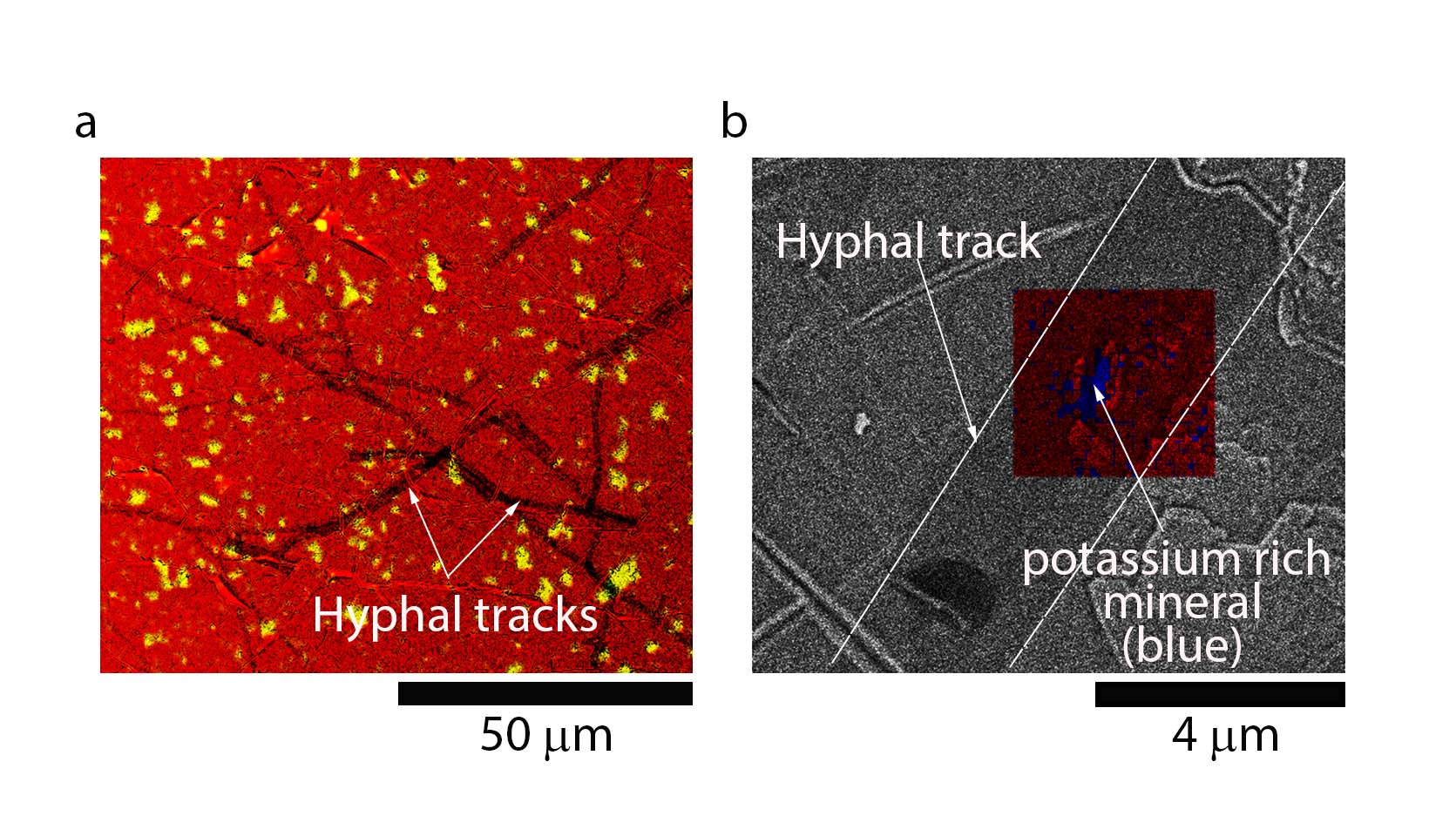

Supplement: FIG S8 [file msystems.00913-22-s0008.jpg]

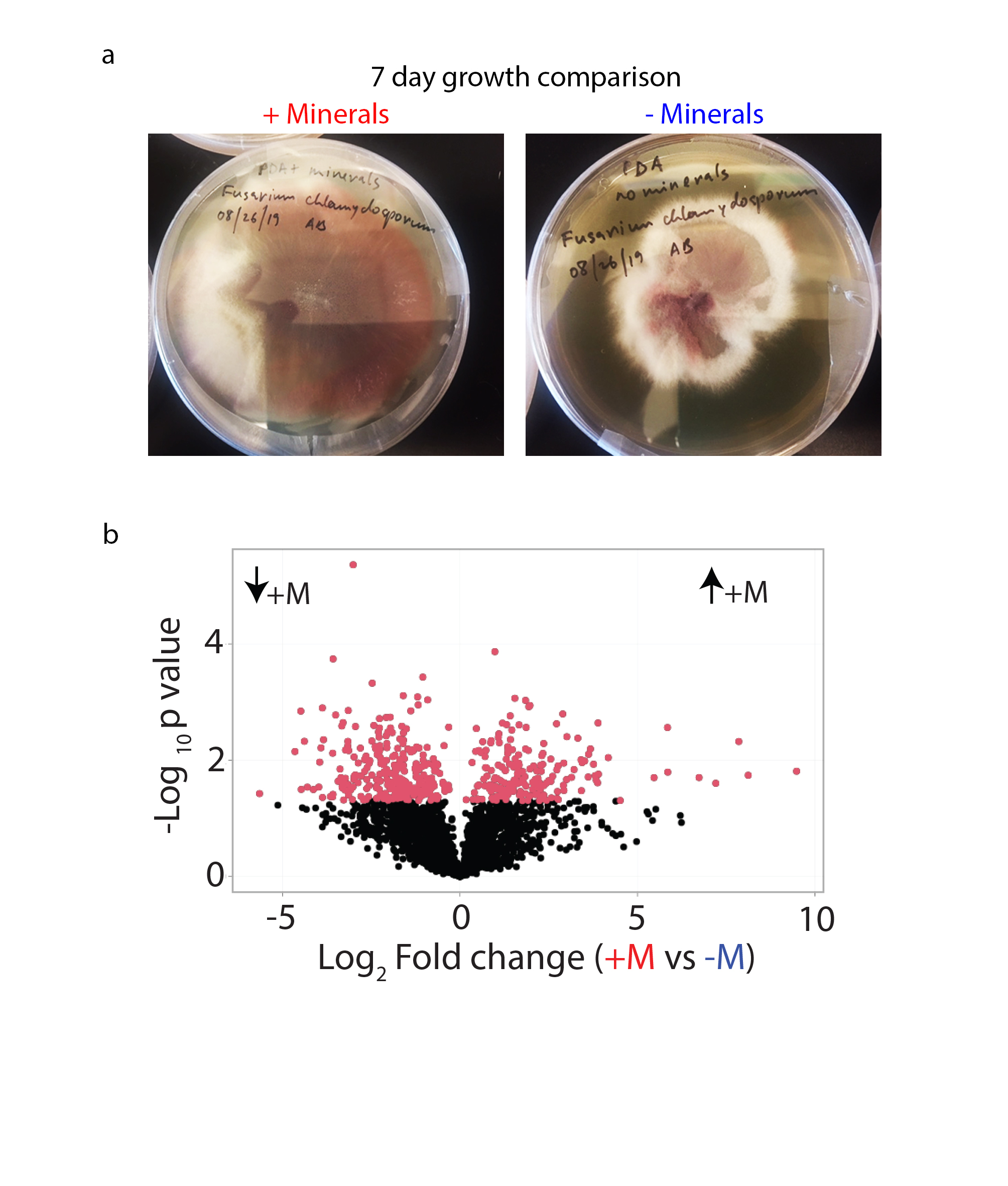

Supplement: FIG S9 [file msystems.00913-22-s0009.jpg]

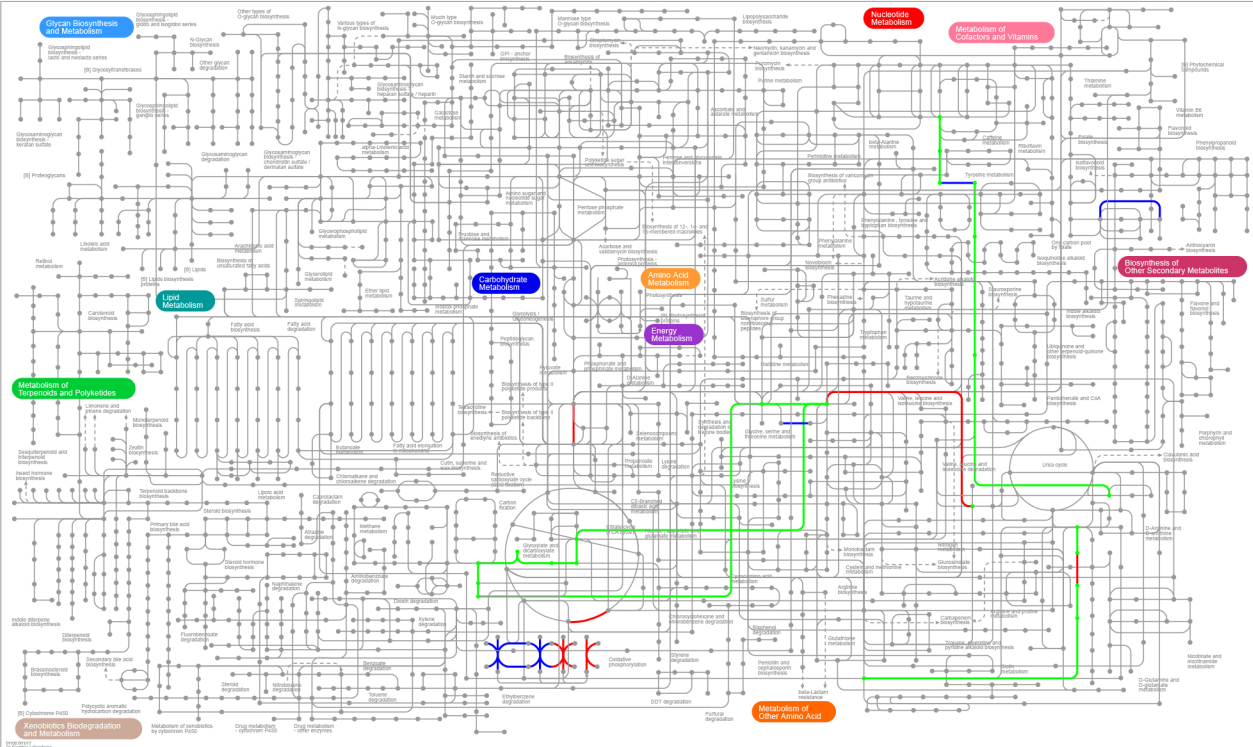

Supplement: FIG S10 [file msystems.00913-22-s0010.pdf]
